# Supplementary figures and images for: Quantifying plasmid movement in drug-resistant Shigella species using phylodynamic inference
Source: PLoS Pathog. 2025 Dec 1;21(12):e1013621. doi: 10.1371/journal.ppat.1013621 (PMC12677775; doi:10.1371/journal.ppat.1013621)

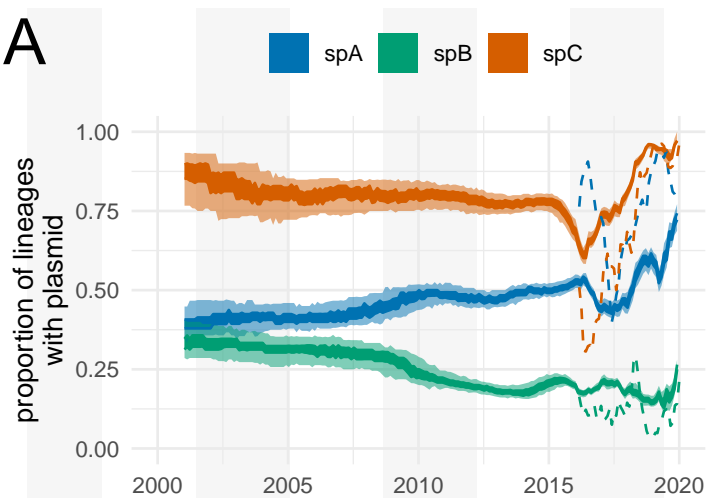

### B

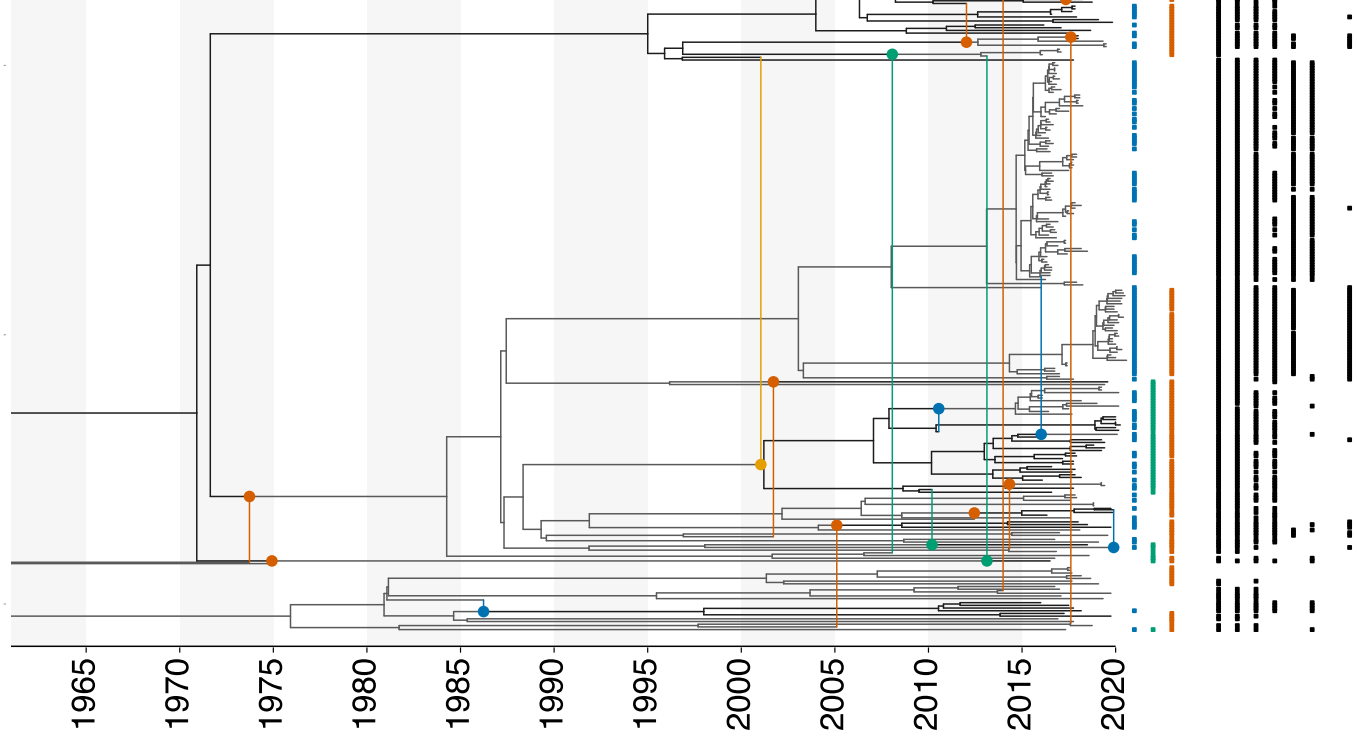

### C

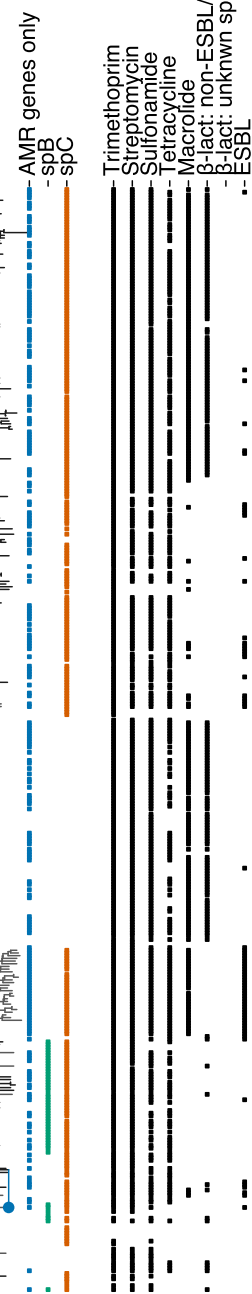

Supplement: S1 Fig — A Proportion of lineages carrying a plasmid between 2000 and 2020. The inner shaded areas denote the 50% HPD, and the outer area is the 95% HPD. The dotted lines denote the proportion of samples with a plasmid. B Here, we show the maximum clade credibility (MCC) network of Shigella sonnei inferred using the chromosomal DNA, the virulence plasmid pINV and the small plasmids spA, spB, and spC. Vertical lines are used to denote plasmid transfer events, where the circles denote the branch to which a plasmid was transferred. The color of the circle denotes either spA, spB, or spC, having jumped between bacterial lineages. The dashed lines correspond to branches from which plasmids branch off. The text denotes the posterior probability of plasmid transfer events for events with a posterior support of over 0.5. C The tip labels in blue, green, and red denote if a plasmid was detected at a leaf. The black dots denote the presence of antimicrobial resistance to the antimicrobials on top. (PDF) [file ppat.1013621.s003.pdf]

Inferred number of  
plasmid transfer events

40  
30  
20  
10  
0

entire spA

strAB + sul  
+ flanking

AMR genes  
only

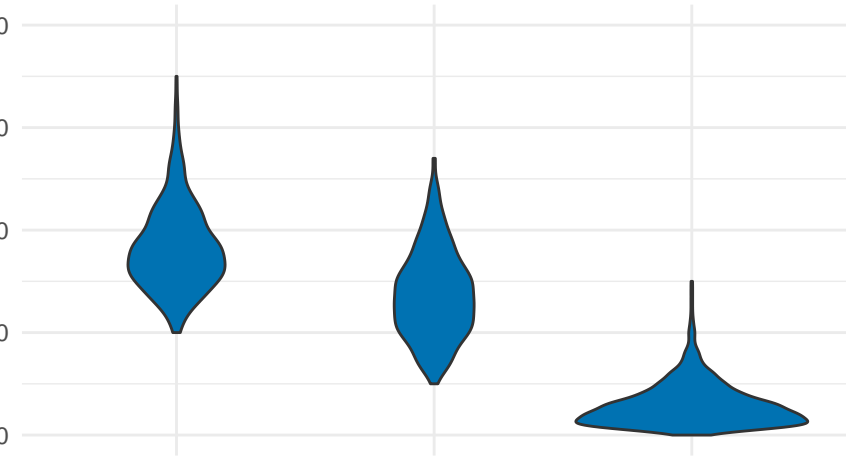

Supplement: S2 Fig — Here, we compare the posterior distribution of the number of plasmid transfer events when using different parts of the spA plasmid for inference. Each violin plot is created from a different analysis using either the entire spA plasmid, the combination of four AMR genes from sul2 to tetA, the three AMR genes sul2, strA, strB, and the flanking region of ∼100 bases. (PDF) [file ppat.1013621.s004.pdf]

A

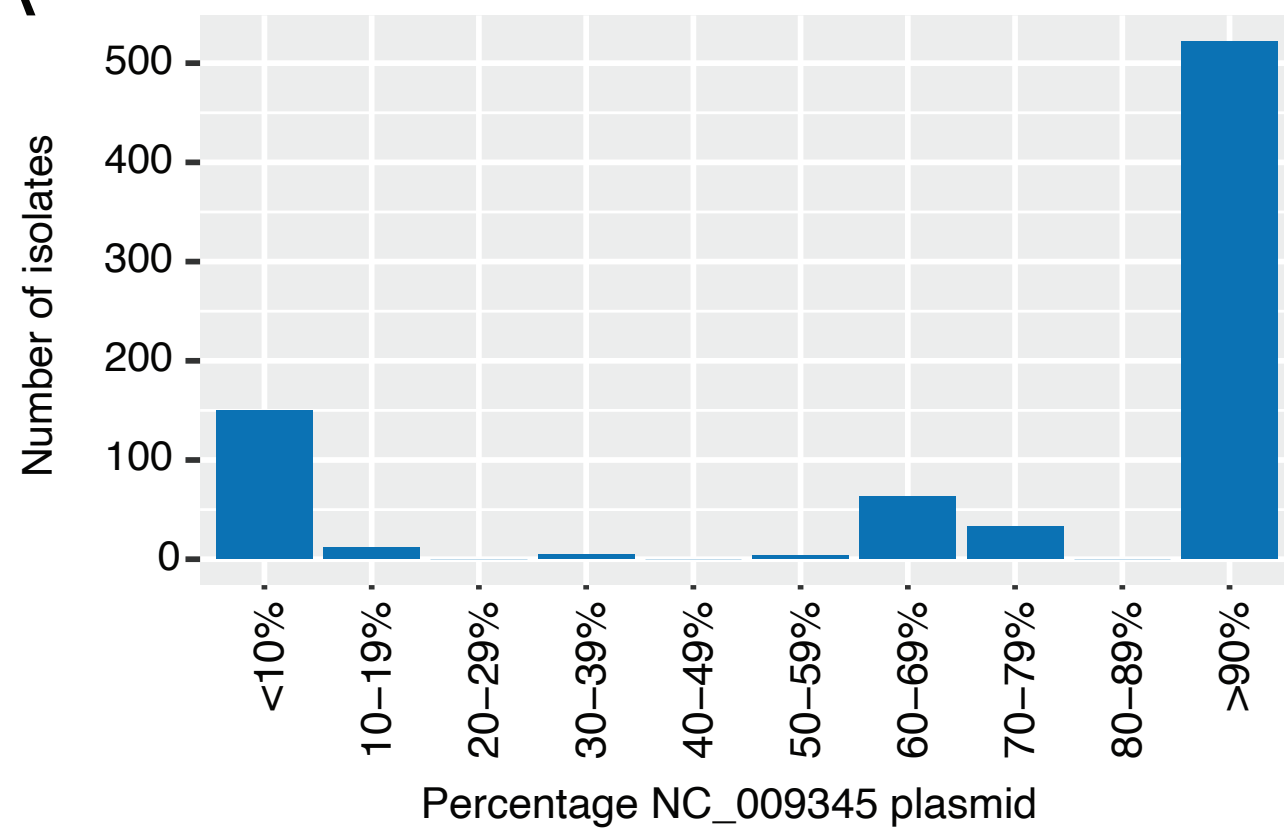

B

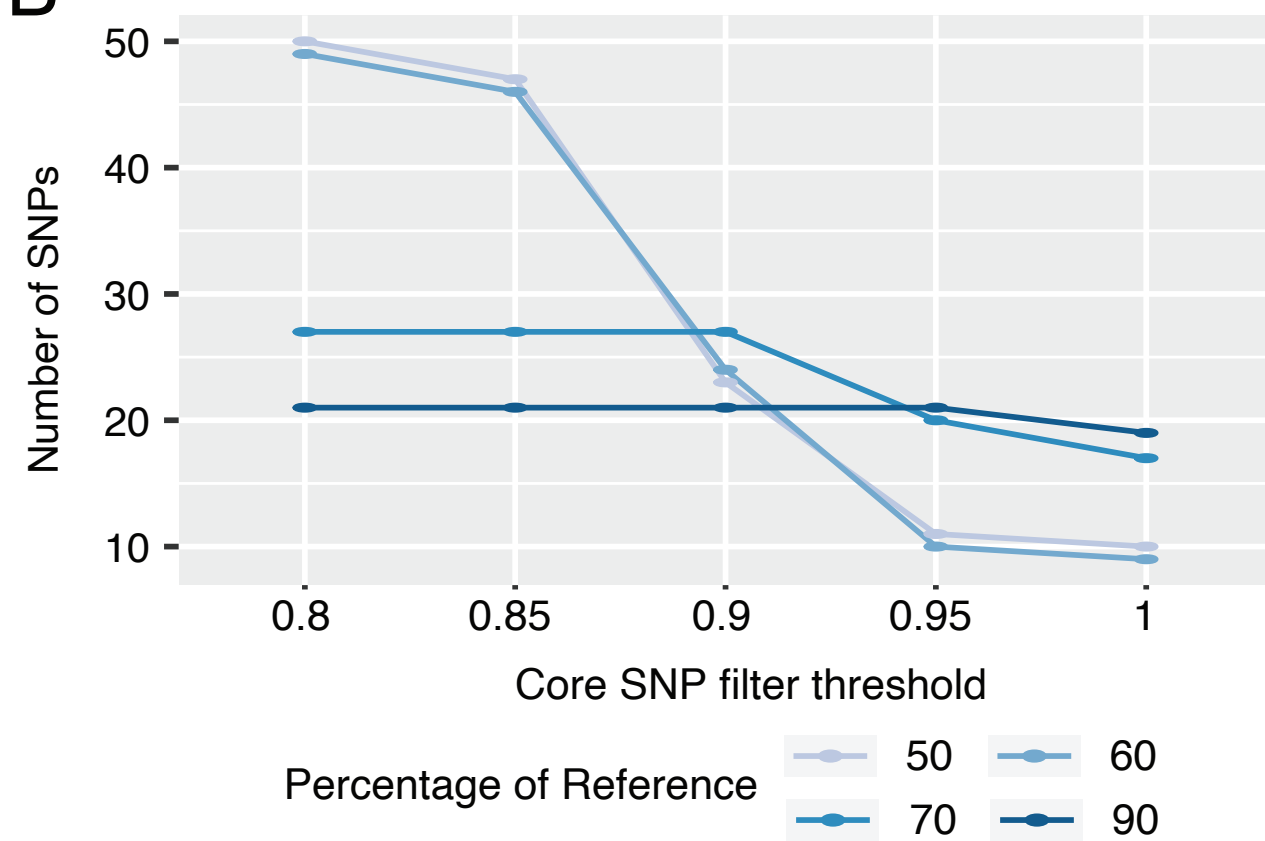

C

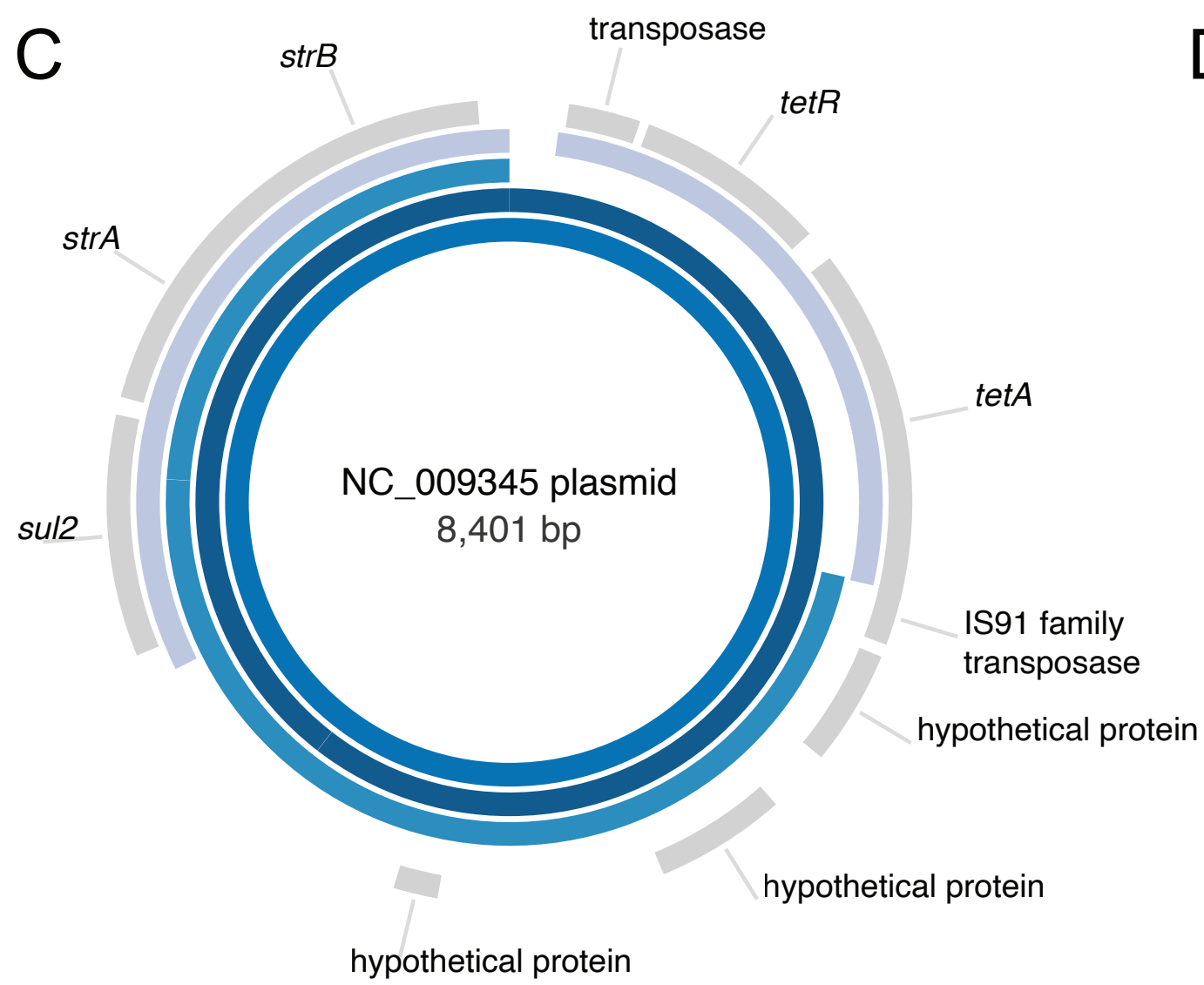

D

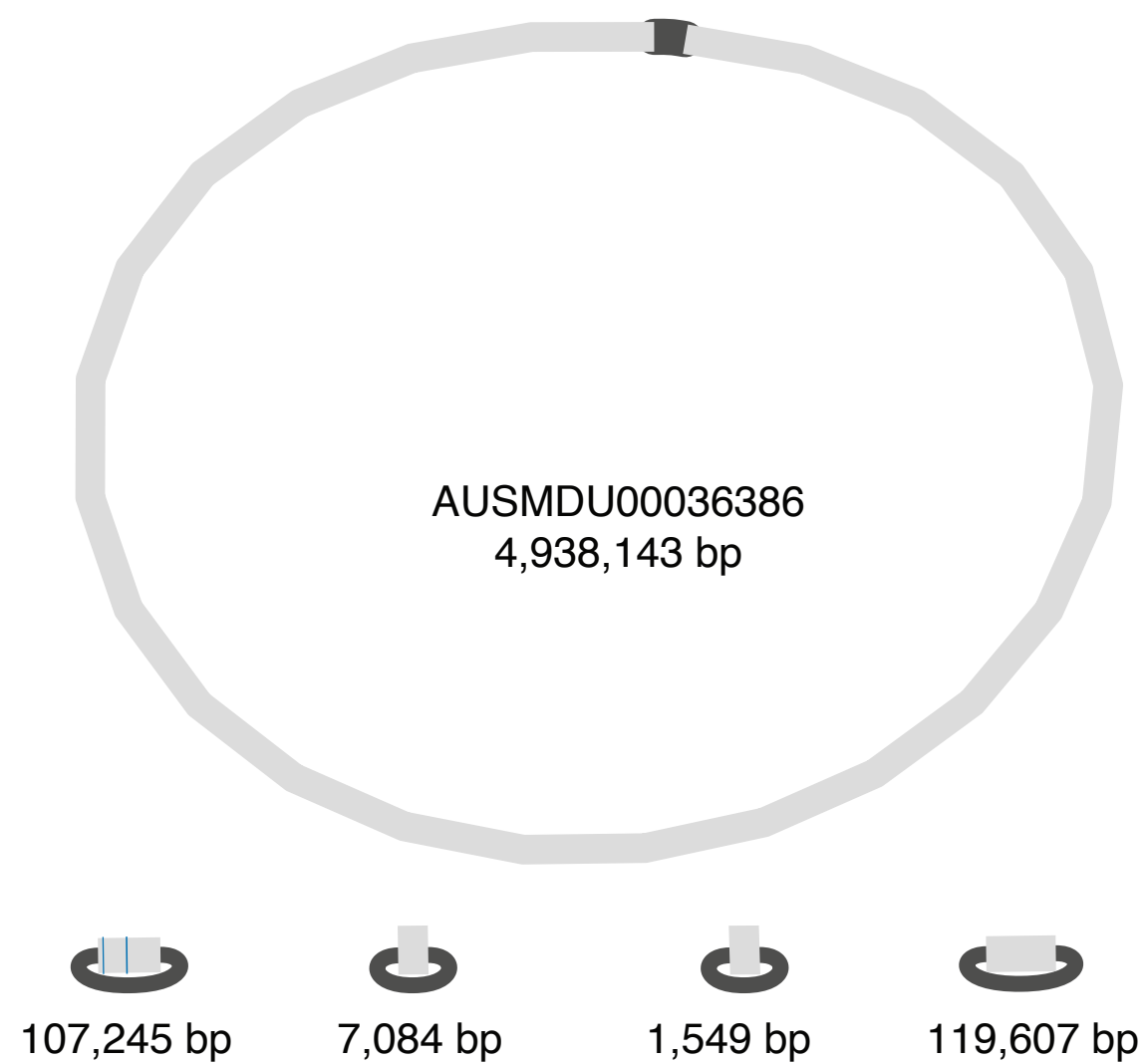

Supplement: S3 Fig — Here, we show the approach for the spA alignment. Panel A) shows the percentage cover of the reference plasmid. Panel B) shows the number of SNPs detected at four different percentages of reference thresholds and different core SNP filter thresholds. Panel C) shows the visualization of contigs from ONT assemblies for representative isolates AUSMDU00029307 (≥90), AUSMDU00020566 (≥70), and AUSMDU00036386 (≥50) at different percentage cover of the reference plasmid. Panel D) the complete genome assembly of AUSMDU00036386, which shows a single chromosome and four plasmids. The blue lines in the plasmid of 107,245 bases show the blast hits for the two AMR regions in spA. (PDF) [file ppat.1013621.s005.pdf]

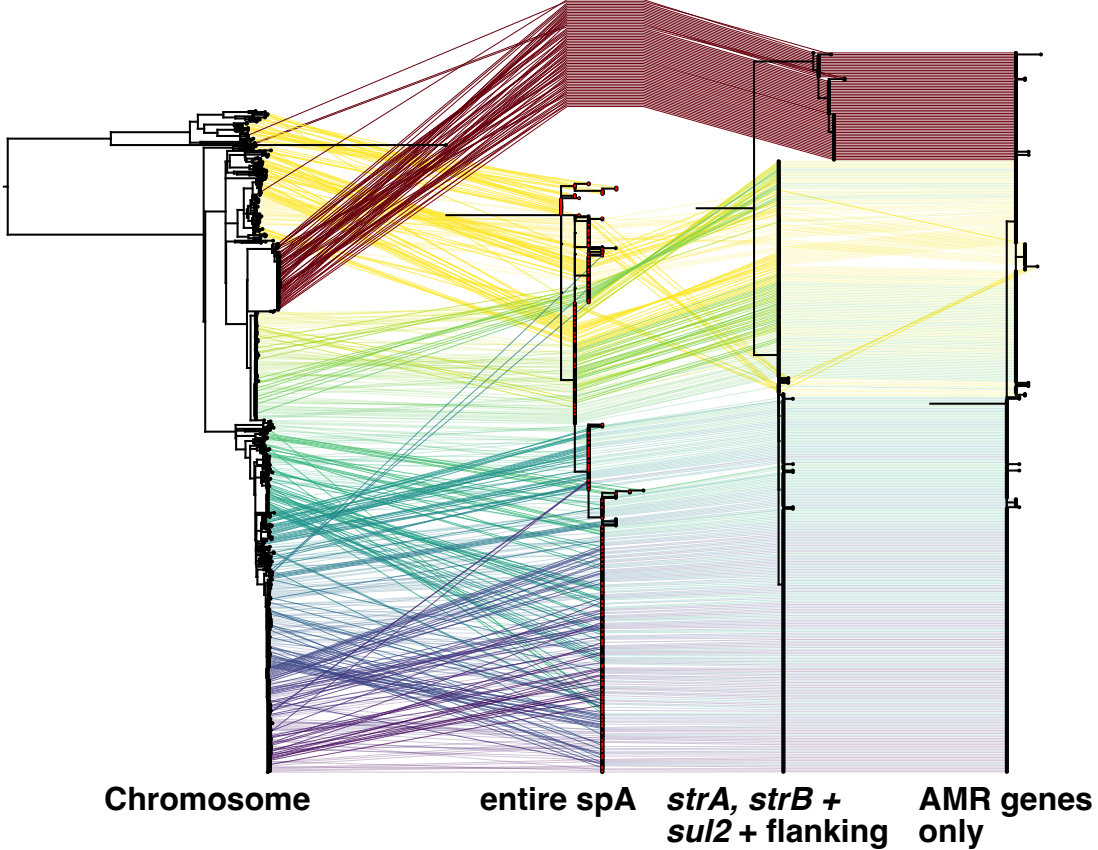

Supplement: S4 Fig — Here, we show a tangle-gram of four trees. The leftmost tree represents the chromosomal data, and the next tree the corresponding spA sequences with more than 70% coverage of the reference genome. When using the 70% coverage threshold, the clade denoted by the red line is removed from the dataset. (PDF) [file ppat.1013621.s006.pdf]

# Reassortment Node Count Distribution

Frequency

0

5

10

15

20

Reassortment Node Count

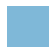

posterior

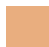

prior

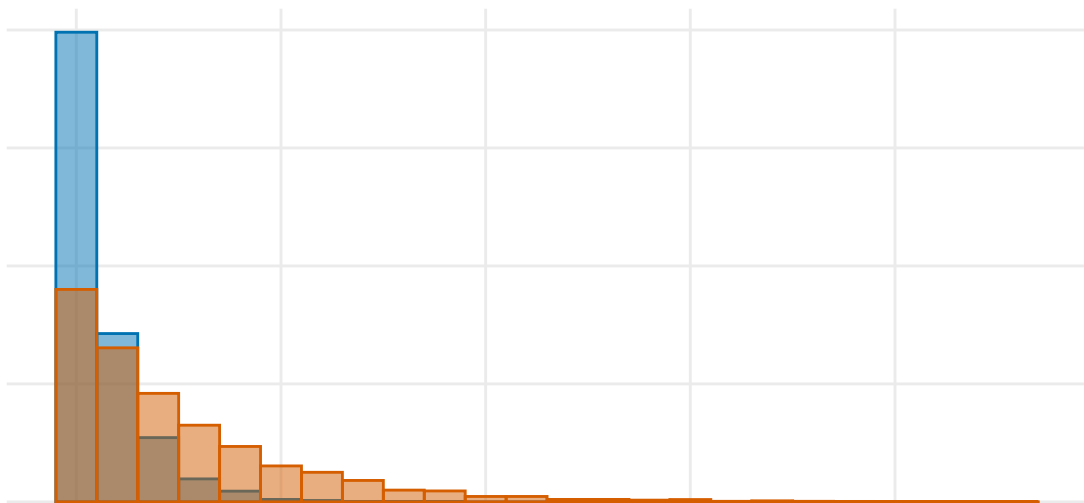

Supplement: S5 Fig — Here, we use Bayesian inference to test for a signal of different trees between the different spA alignments used. To do so, we ran a coalescent with reassortment analyses using the three spA alignments as segments. We used an exponential with a mean of 0.01 as a prior on the reassortment rates. We used the coalescent with reassortment over plasmid transfer, as there is no main segment for this analysis. We then show the expected counts for the reassortment event under the prior compared to the inferred counts. As shown, the data between the three spA alignments increases the weight of zero events, showing an absence of signal for the different alignments to code for different evolutionary histories (PDF) [file ppat.1013621.s007.pdf]

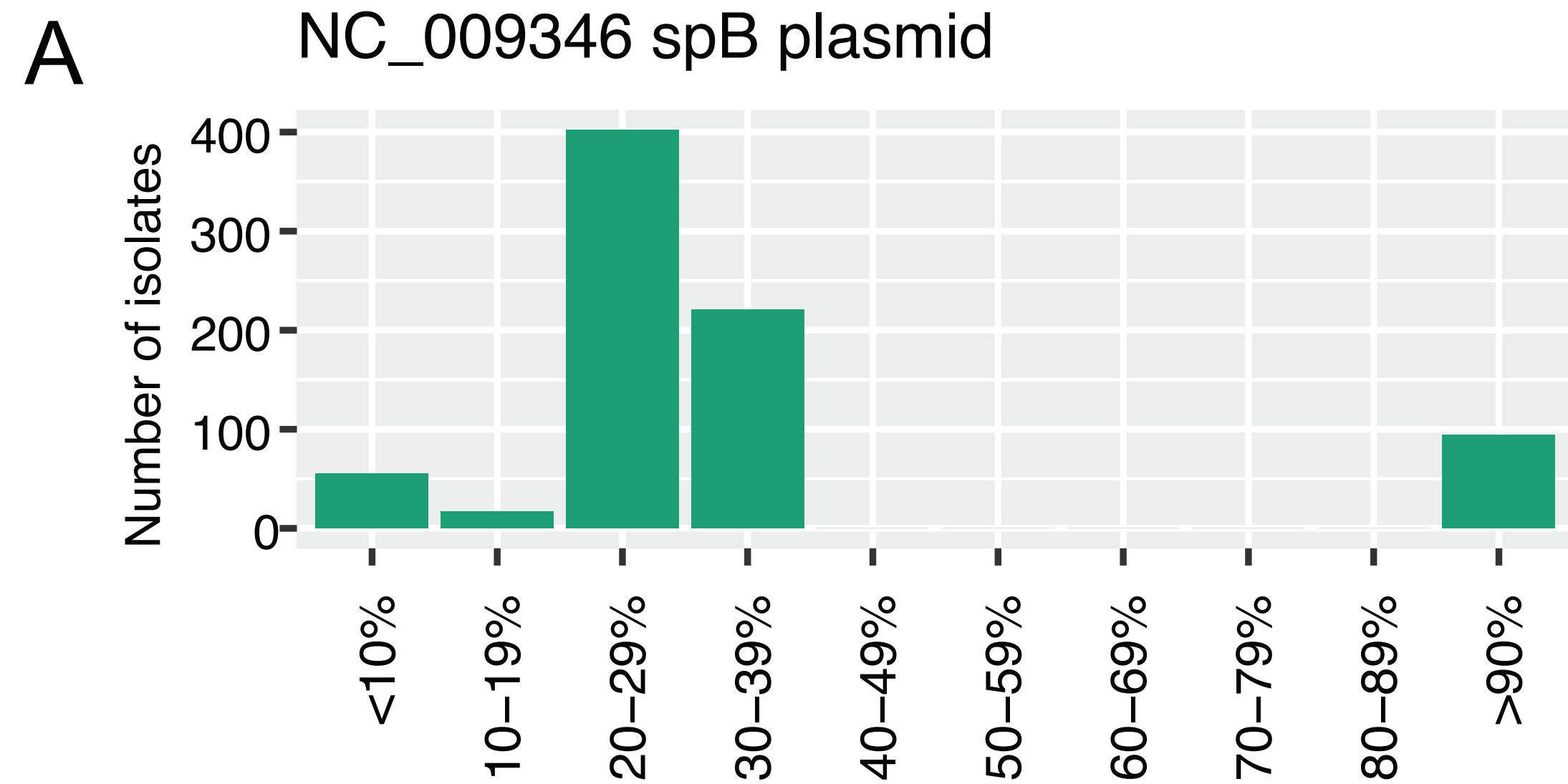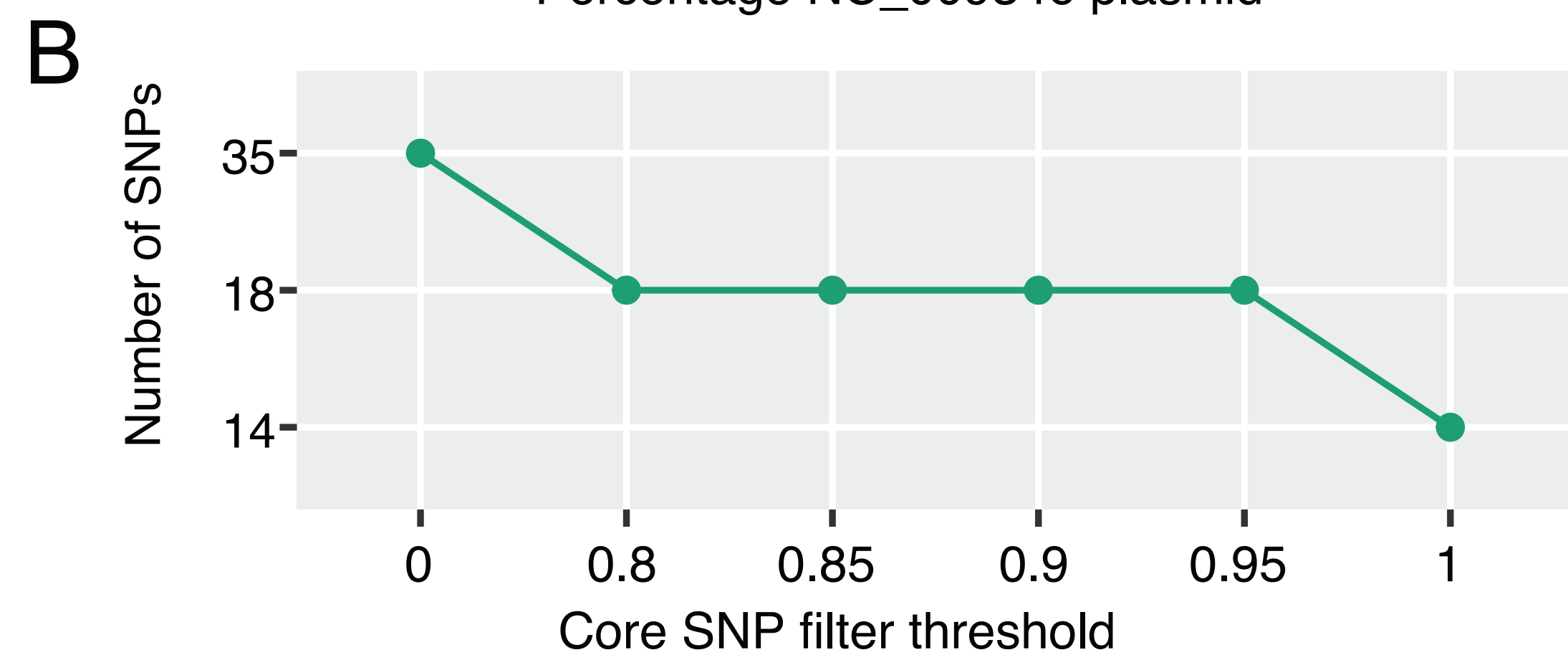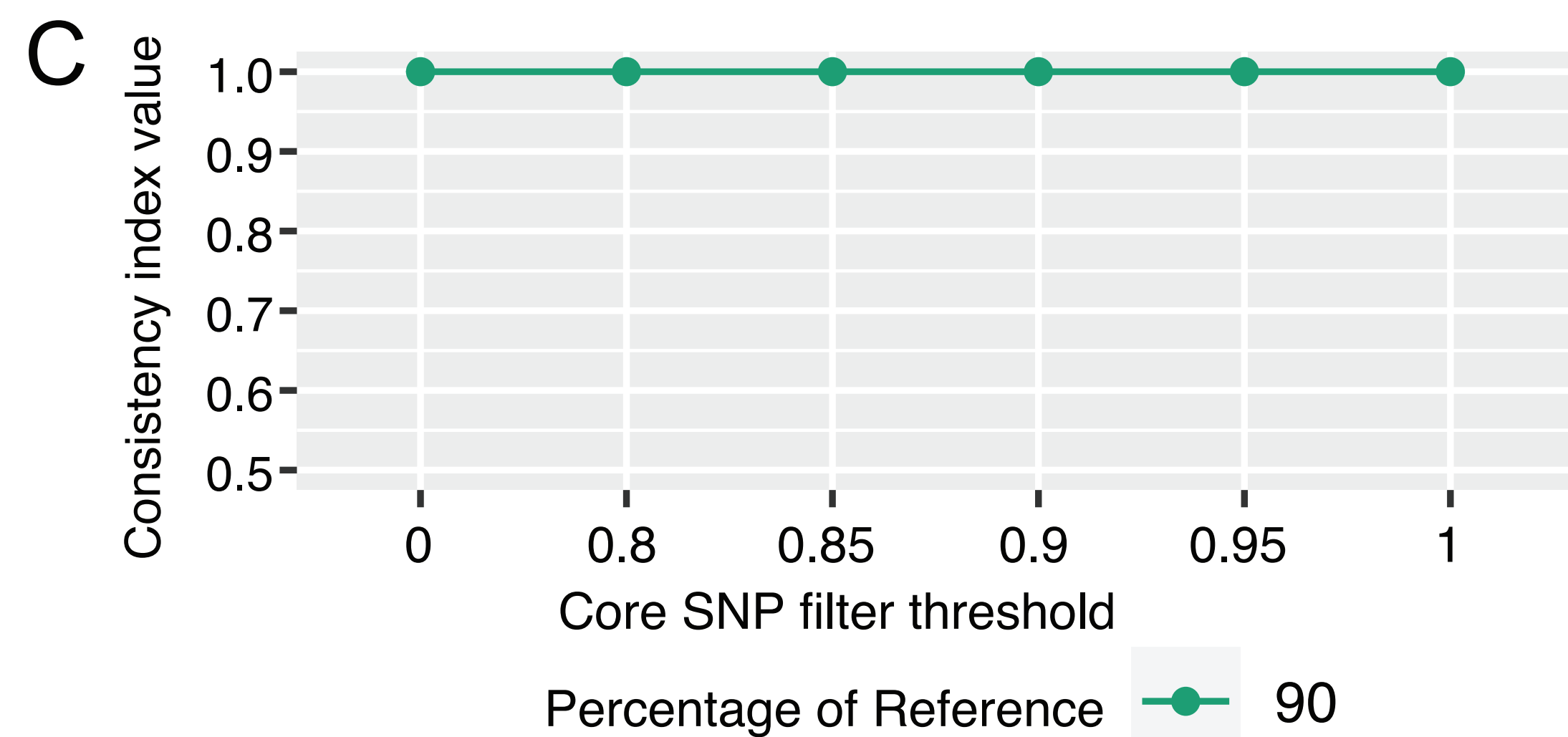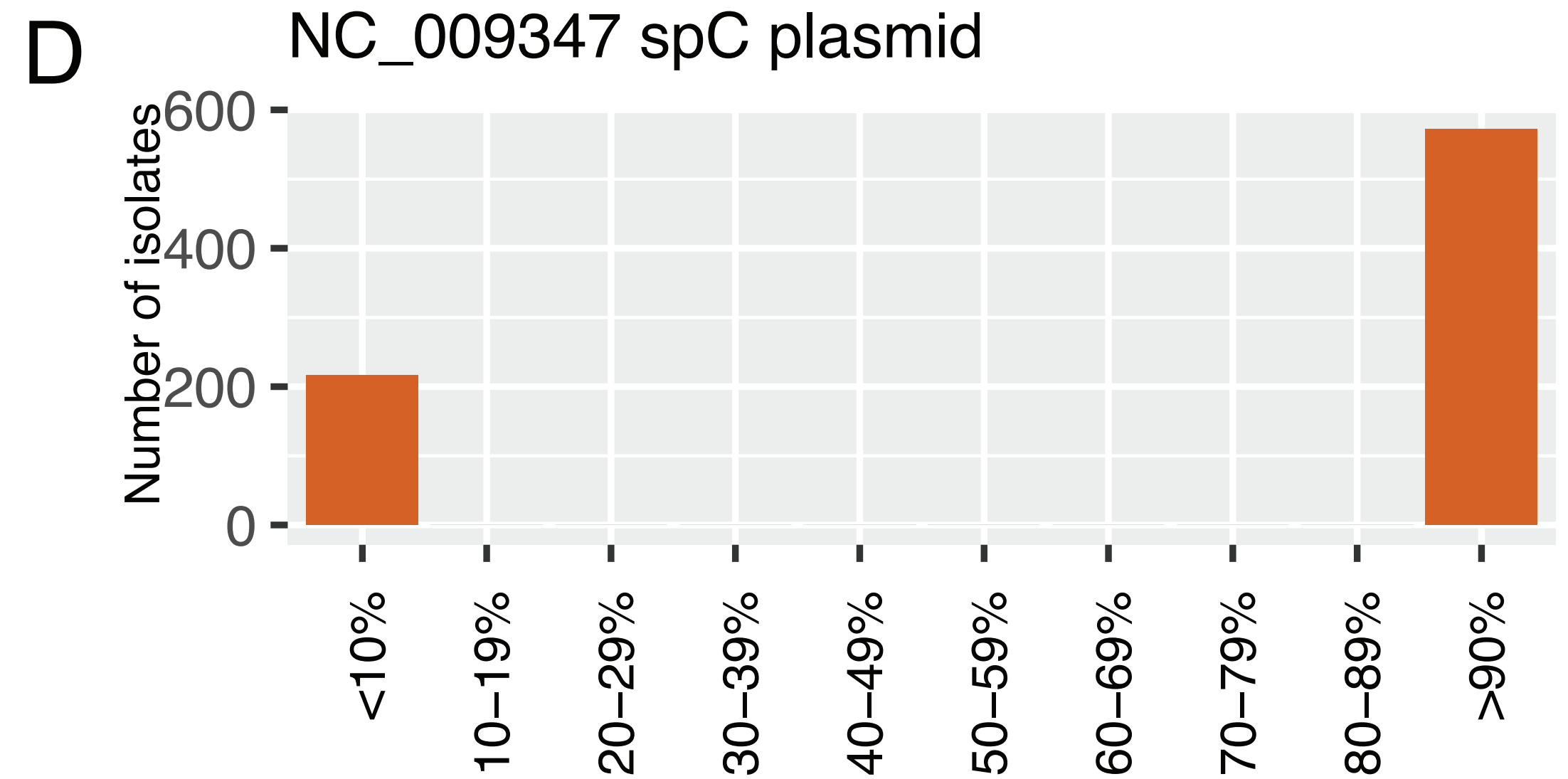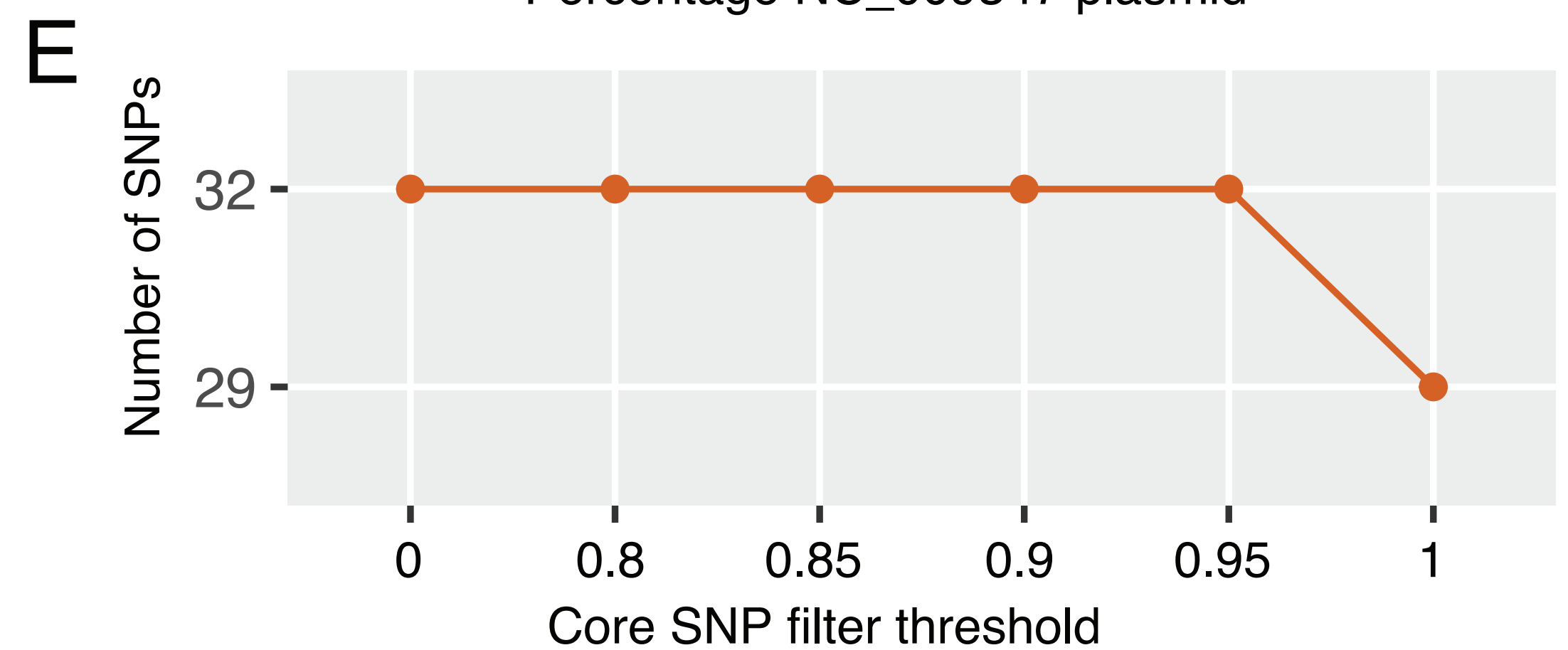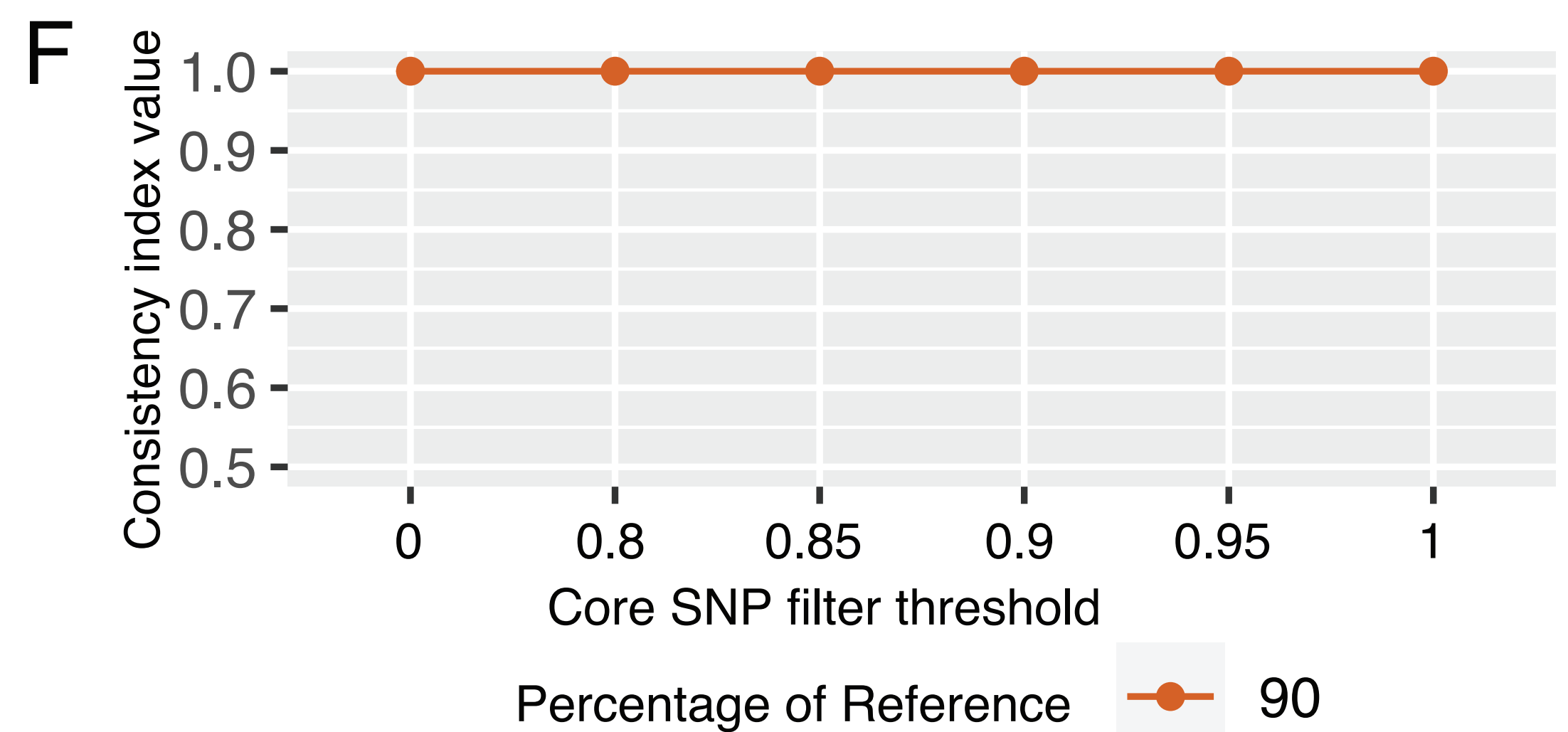

Supplement: S6 Fig — Here, we show the approach for the spB and spC alignment. Panels A) and D) show the percentage cover of the reference plasmid for spB and spC, respectively. Panels B) and E) show the number of SNPs detected in each dataset for isolates with ≥90 of the plasmid reference with different core SNP filter thresholds. Panels C) and F) show the consistency index (CI) at different core SNP thresholds. (PDF) [file ppat.1013621.s008.pdf]

Plasmid transfer rate per year

0.05  
0.04  
0.03  
0.02  
0.01  
0.00

entire spA

strA & B + sul + flanking

AMR genes only

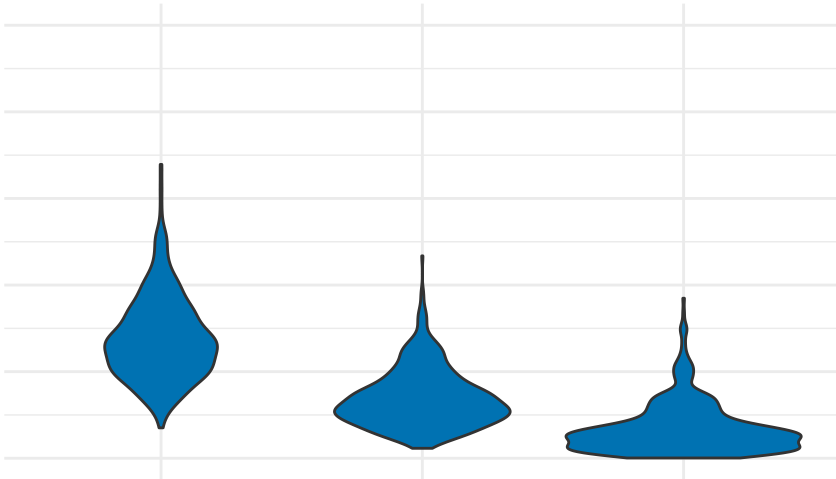

Supplement: S9 Fig — Here, we compare the posterior distribution of the plasmid transfer rate, focusing only on spA and comparing the use of different parts of the spA plasmid for inference. Each violin plot is created from a different analysis using either the entire spA plasmid, the combination of four AMR genes from sul2 to tetA, the three AMR genes sul2, strA,strB, and the flanking region of ∼100 bases. (PDF) [file ppat.1013621.s011.pdf]

# Rate of change in plasmid presence absence

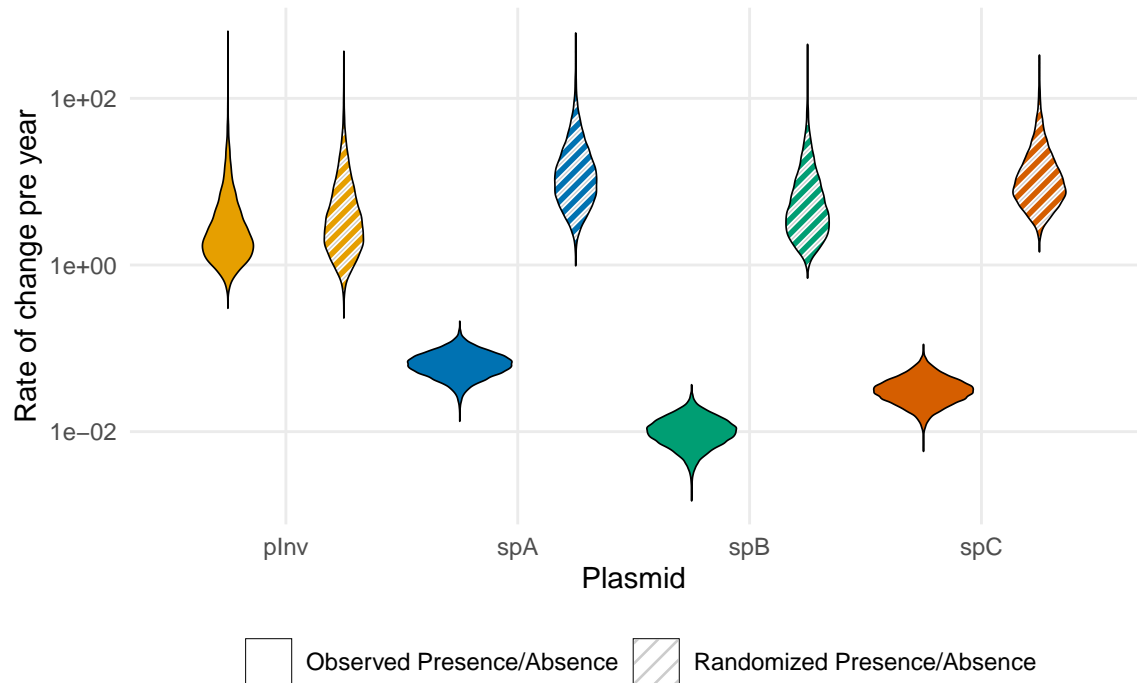

Supplement: S11 Fig — Here, we model the presence/absence of a plasmid at the tips using a continuous-time Markov chain. For each plasmid, we use once the actual observed presence/absence data and once a permutated tip to plasmid assignment. If the plasmid were always present, but is randomly lost during the sampling procedure, such as in culture, we would expect no difference between the gain and loss rates of plasmid for the true and permutated data. If there is substantial inherited structure in the presence or absence of plasmids, we would expect to estimate substantially lower rates in the true compared to the randomized tip to plasmid presence assignment. (PDF) [file ppat.1013621.s013.pdf]

# Posterior Estimates of Plasmids being gained or lost

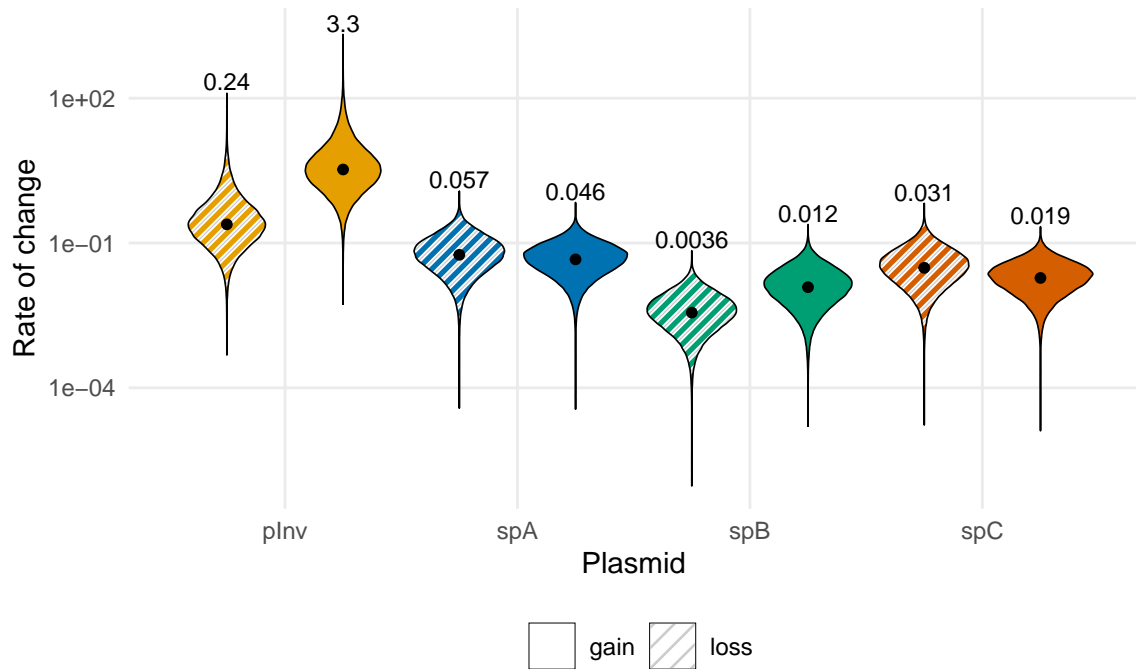

Supplement: S12 Fig — We infer the rates of gain and loss of pINV, spA, spB, and spC using a discrete trait model where we model the presence and absence of each plasmid as a continuous-time Markov chain. The rates show the posterior estimates, and the dots and numeric values show the median estimate. (PDF) [file ppat.1013621.s014.pdf]

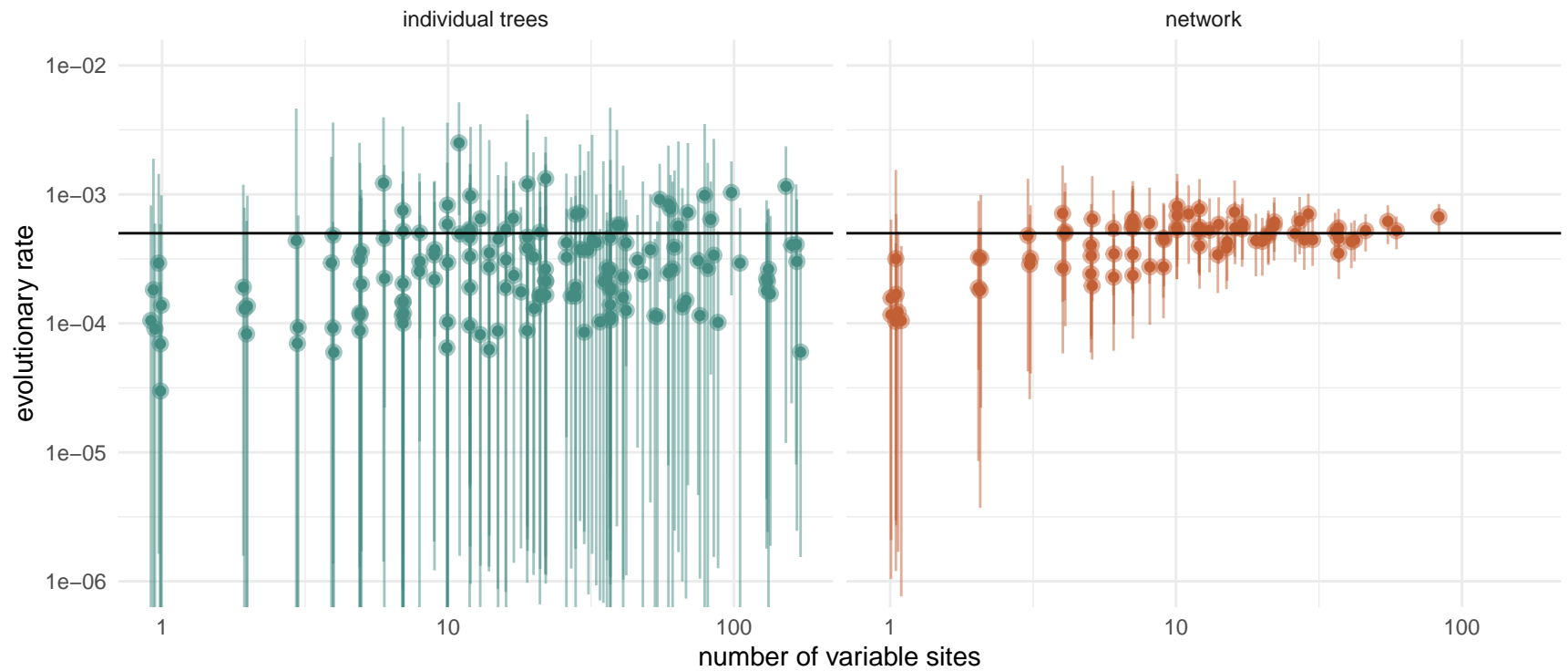

Supplement: S13 Fig — We simulated 50 phylogenetic networks under the coalescent with plasmid transfer with three plasmids sampled over five years. We assume that the chromosome and the three plasmids evolved at a rate of 5×10^−4^ subs/site/unit time. The chromosome has an SNP alignment length of 8000bp, while the three plasmids had SNP alignments of 200bp, 100bp, and 50bp, respectively. These settings will produce approximately the same number of SNPs per unit of time as a chromosome of 4.8 Mbp evolving at a rate of 8×10^−7^ subs/site unit time. On the y-axis, we show the inferred evolutionary rates with the error bars denoting the 95% HPD and the point denoting the mean estimates. The x-axis is the number of variable sites in the alignment. (PDF) [file ppat.1013621.s015.pdf]

# Coefficient of Variation for true and simulated data

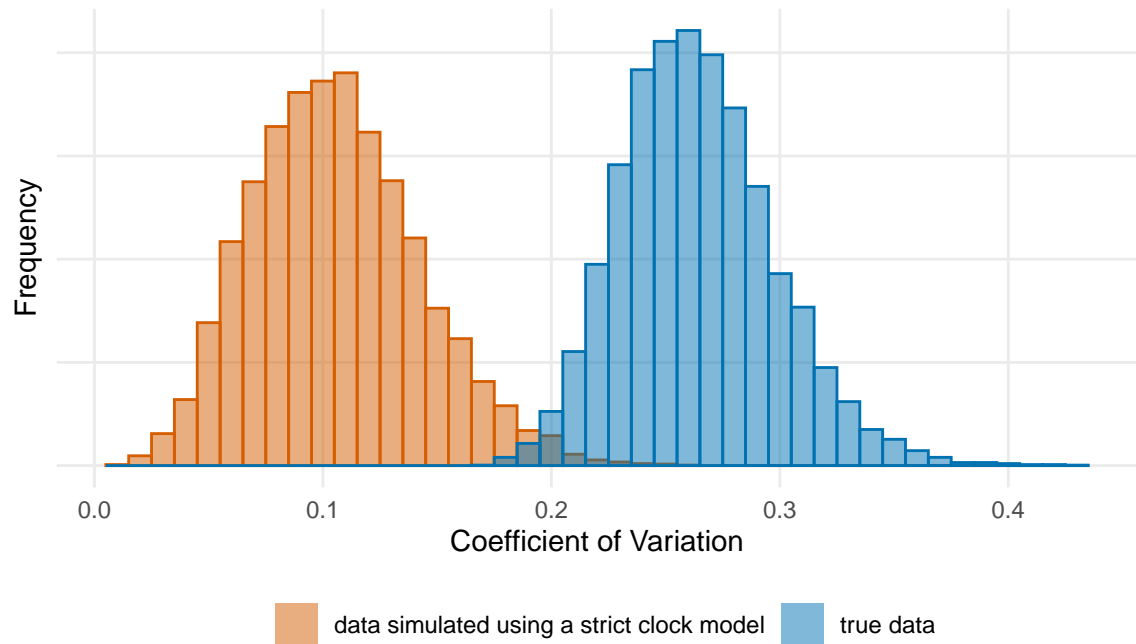

Supplement: S15 Fig — Here, we inferred the evolutionary history of S. sonnei using 400 random isolates of the chromosome sequences using a log-normal relaxed clock model in BEAST2 and a constant coalescent prior. We then simulate an alignment using the mean clock rates on top of a random tree in the posterior and a strict clock model. We next re-inferred the evolutionary history in the case where we know that the true clock model was a strict clock model and compared the coefficient of variation between the true data and the data simulated under a strict clock model. The true data shows some signal for rate variation across, however, the intervals are slightly overlapping, indicating that there is not substantial rate variation. (PDF) [file ppat.1013621.s017.pdf]

A

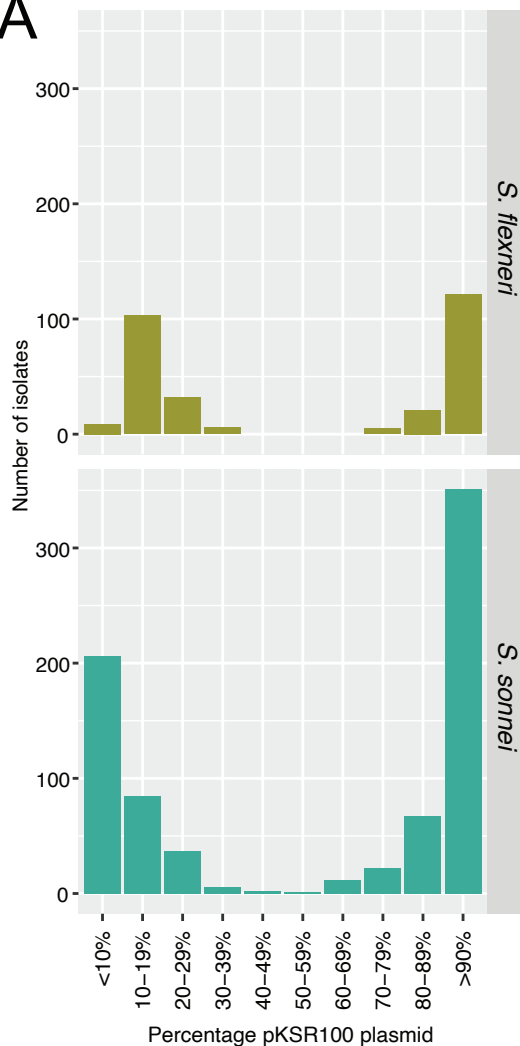

B

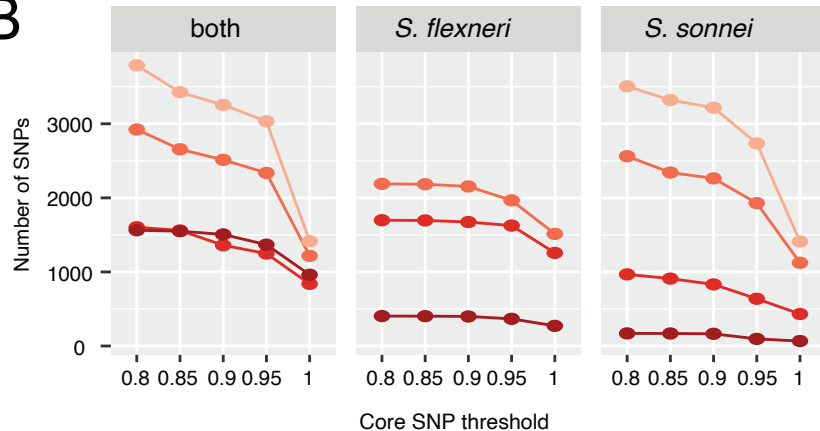

C

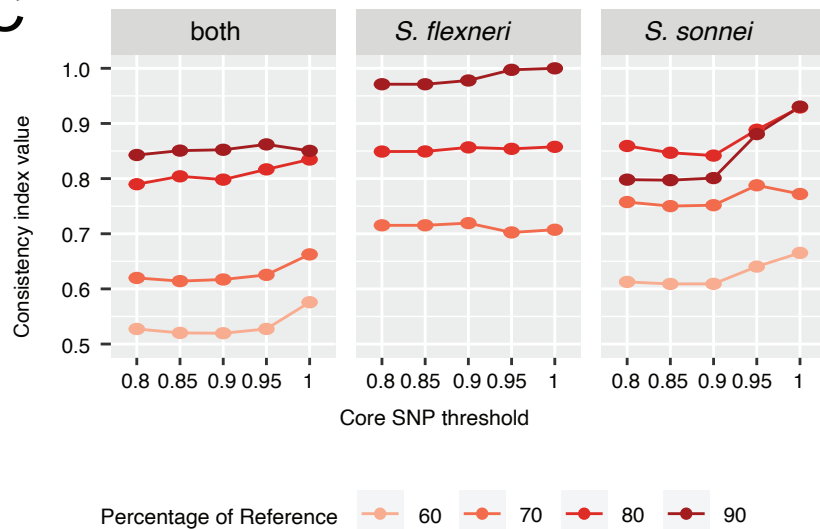

Supplement: S16 Fig — Here, we show the approach for the MDR plasmid pKSR100 alignments. Panel A) shows the percentage cover of the reference pKSR100 plasmid for S. sonnei and S. flexneri. Panel B) shows the number of SNPs detected in each dataset for isolates with four different thresholds for the coverage of the plasmid reference with different core SNP filter thresholds. Panels C) show the consistency index (CI) of four different thresholds for the coverage of the plasmid reference with different core SNP filter thresholds for three datasets. (PDF) [file ppat.1013621.s018.pdf]

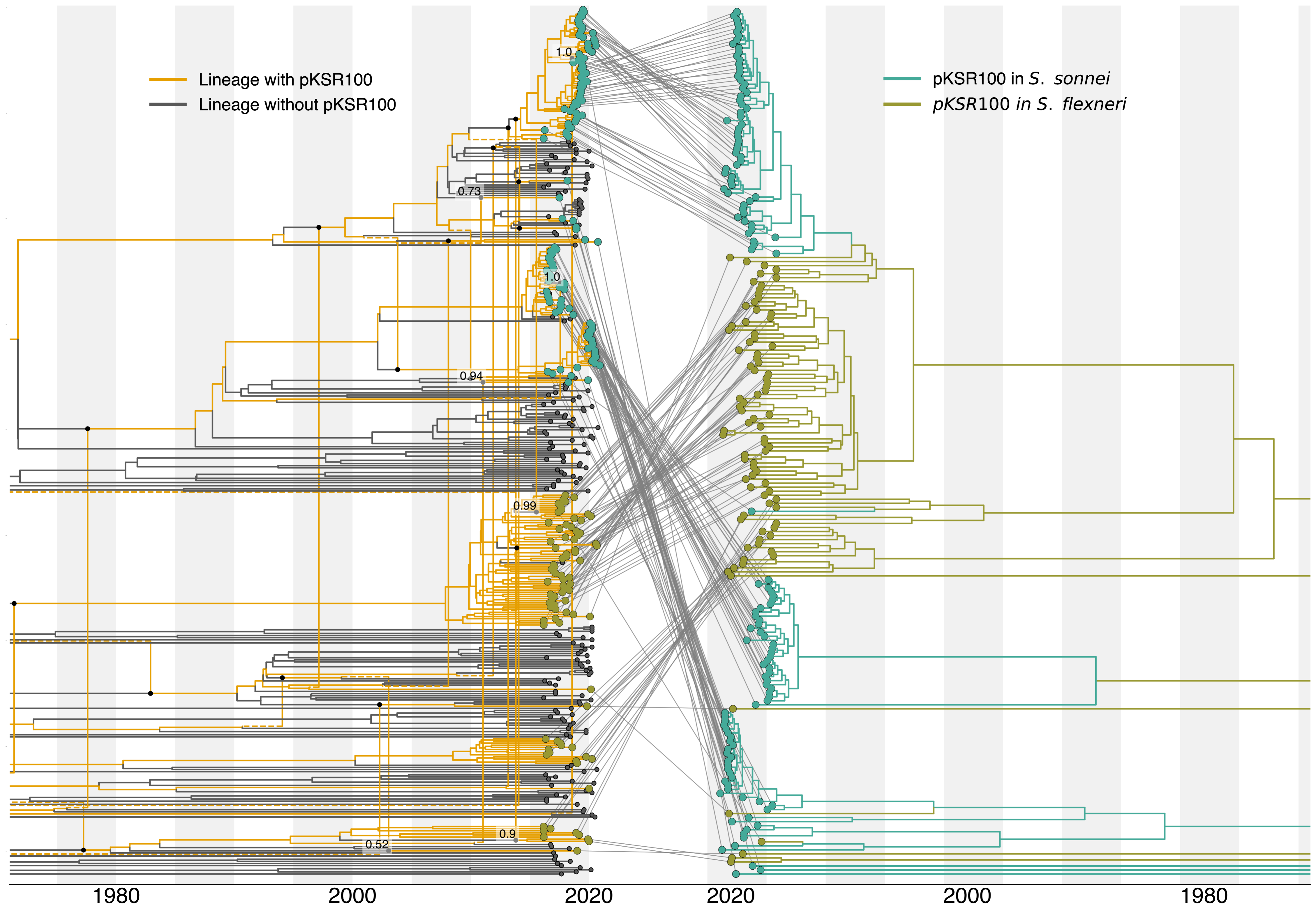

Supplement: S17 Fig — MCC network of S. sonnei and S. flexneri samples with the embedding of the pKSR100 plasmid tree A. The text denotes the posterior support values for plasmid transfer events. B Plasmid tree of pKSR100 with the host species S. sonnei or S. flexneri mapped onto the tree. The different colors of the tips show clusters of sequences that are the result of separate introductions of the MDR plasmid. MCC: maximum clade credibility. MDR: multidrug resistance (PDF) [file ppat.1013621.s019.pdf]

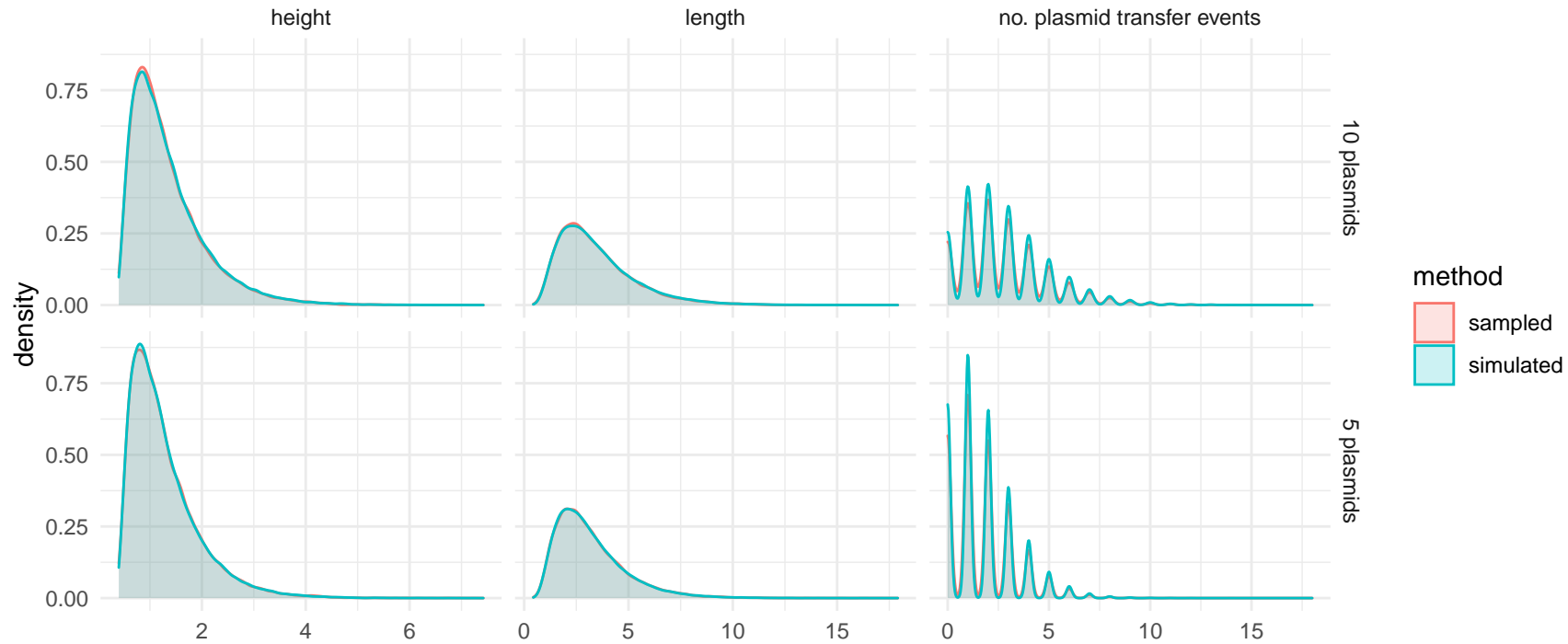

Supplement: S18 Fig — To validate the implementation of CoalPT, we simulated networks under the CoalPT model, once with 5 plasmids and once with 10 plasmids. We then sampled phylogenetic networks under our implementation of the CoalPT model in BEAST2 under the prior (i.e., without any sequence information). As shown here, the summary statistics between networks simulated and sampled (using MCMC) under CoalPT match. (PDF) [file ppat.1013621.s020.pdf]

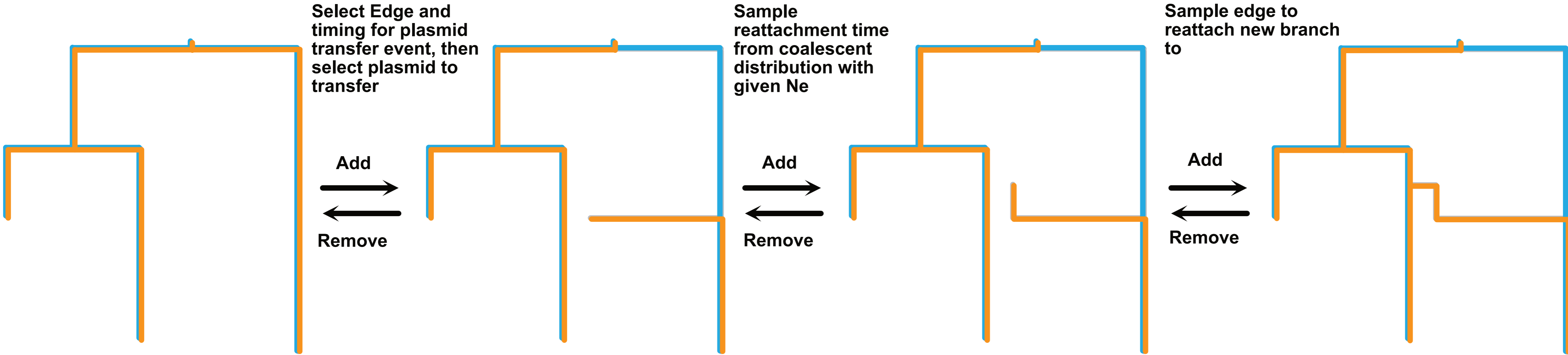

Supplement: S19 Fig — The add remove operator selects a random edge to add a plasmid transfer event to. On that edge, a random time is selected and a random plasmid is ’transferred’. We then sample the time to the next coalescent event under the constant coalescent, which becomes the reattachment time. Lastly, we select a random edge to reattach the plasmid edge to. (PDF) [file ppat.1013621.s021.pdf]

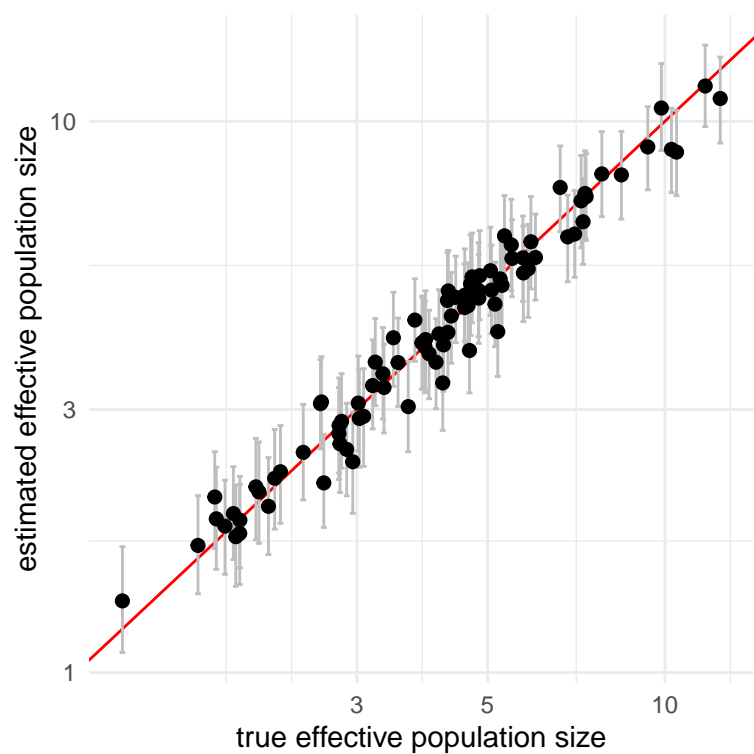

Supplement: S20 Fig — To test the performance of the coalescent with plasmid transfer, we simulated 100 networks in a well-calibrated simulated study. The effective population sizes were sampled from a Lognormal distribution with M=1.4844 and S=0.5. The plasmid transfer rates were sampled from a Lognormal distribution with M=-1.7344 and S=0.5. We then simulated genomic sequences for the core genome and 3 plasmids under the Jukes-Cantor Model. Last, we inferred the phylogenetic network, effective population sizes, and plasmid transfer rates from these sequences using the above lognormal distributions as priors on the Ne and plasmid transfer rates. Here, we show the inferred Ne sizes (y-axis) compared to simulated Ne (x-axis). The point denotes the median estimate and the error bars the lower 95% highest posterior density interval. (PDF) [file ppat.1013621.s022.pdf]

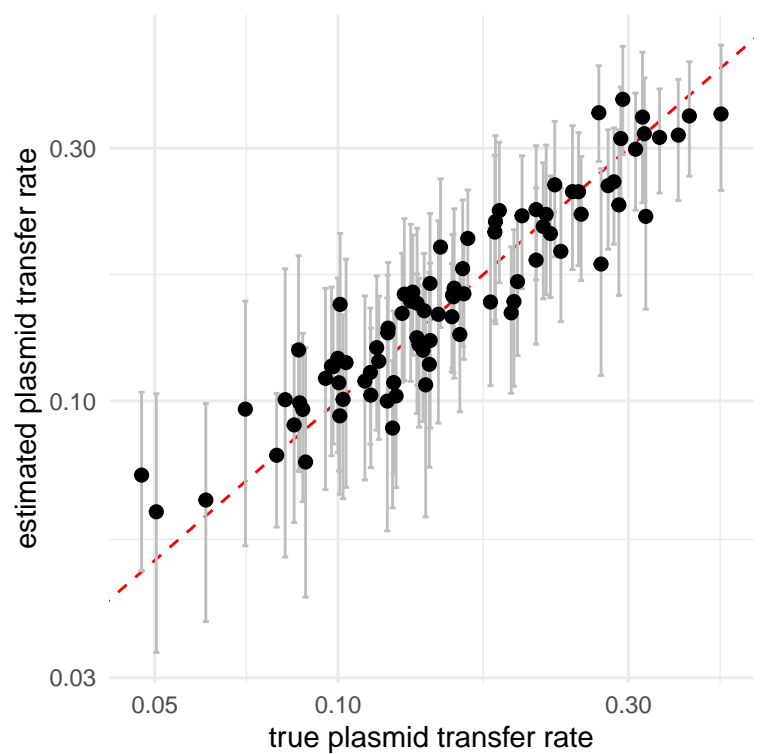

Supplement: S21 Fig — Here, we show the inferred plasmid transfer rates(y-axis) compared to the true/simulated rates on the x-axis. These estimates are from the same analyses as the ones in S20 Fig. The point denotes the median estimate and the error bars the lower 95% highest posterior density interval. (PDF) [file ppat.1013621.s023.pdf]
