# Supplementary material for: Quantifying plasmid movement in drug-resistant Shigella species using phylodynamic inference
Source: PLoS Pathog. 2025 Dec 1;21(12):e1013621. doi: 10.1371/journal.ppat.1013621 (PMC12677775; doi:10.1371/journal.ppat.1013621)
Supplement: S7 Fig — Here, we show the posterior distribution of how often a pINV (the virulence plasmid), spA, spB, and spC on the x-axis moved between S. sonnei lineages on the y-axis. (PDF) [file ppat.1013621.s009.pdf]

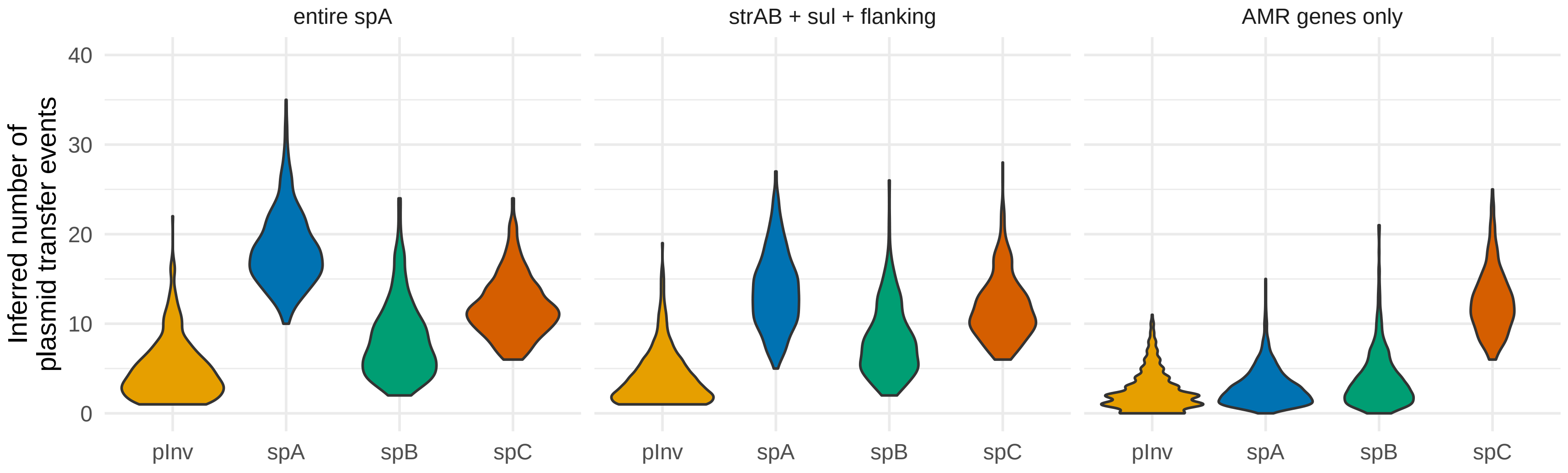
entire spA
strAB + sul + flanking
AMR genes only
Inferred number of
plasmid transfer events
0
10
20
30
40
pInv
spA
spB
spC
pInv
spA
spB
spC
pInv
spA
spB
spC
