# Supplementary material for: Quantifying plasmid movement in drug-resistant Shigella species using phylodynamic inference
Source: PLoS Pathog. 2025 Dec 1;21(12):e1013621. doi: 10.1371/journal.ppat.1013621 (PMC12677775; doi:10.1371/journal.ppat.1013621)
Supplement: S8 Fig — Here, we compare the posterior distribution of the plasmid transfer rate when using different parts of the spA plasmid for inference. Each violin plot is created from a different analysis using either the entire spA plasmid, the combination of four AMR genes from sul2 to tetA, the three AMR genes sul2, strA,strB, and the flanking region of ∼100 bases. (PDF) [file ppat.1013621.s010.pdf]

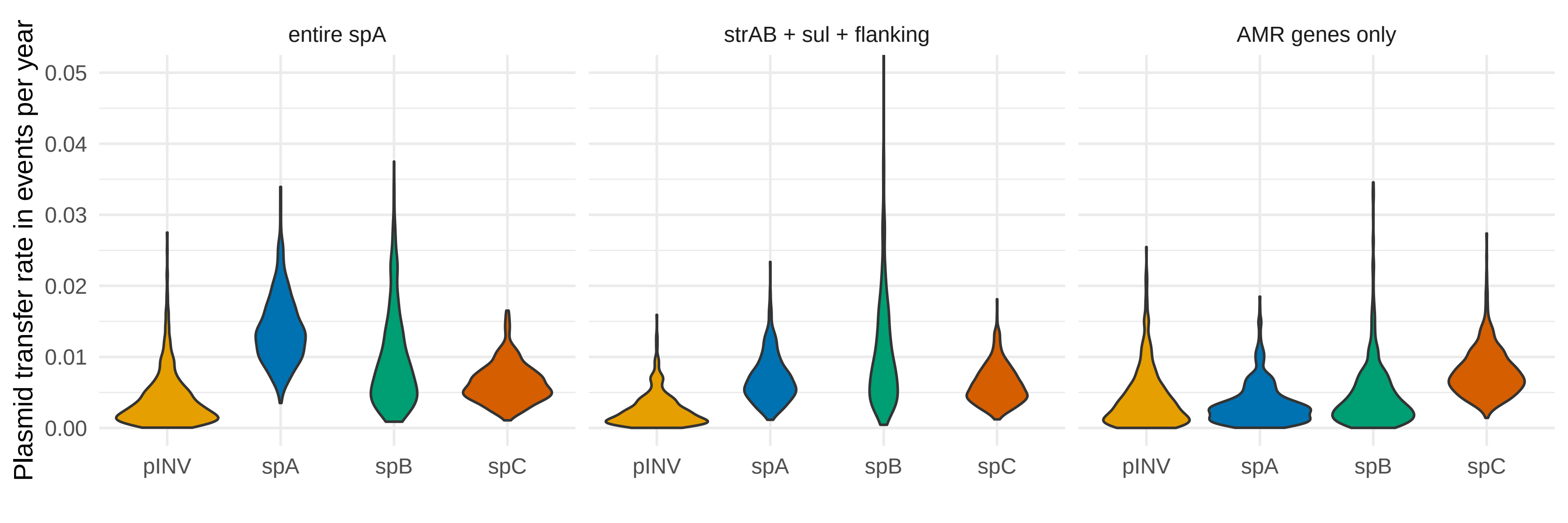
entire spA
strAB + sul + flanking
AMR genes only
Plasmid transfer rate in events per year
0.05
0.04
0.03
0.02
0.01
0.00
pINV
spA
spB
spC
