# Supplementary material for: Quantifying plasmid movement in drug-resistant Shigella species using phylodynamic inference
Source: PLoS Pathog. 2025 Dec 1;21(12):e1013621. doi: 10.1371/journal.ppat.1013621 (PMC12677775; doi:10.1371/journal.ppat.1013621)
Supplement: S10 Fig — We compute the rate at which plasmids are being lost as the number of events where a plasmid was lost divided by the tree length of that plasmid. We assume a plasmid was lost on the edges where the parent node carried a plasmid, while the child node did not. (PDF) [file ppat.1013621.s012.pdf]

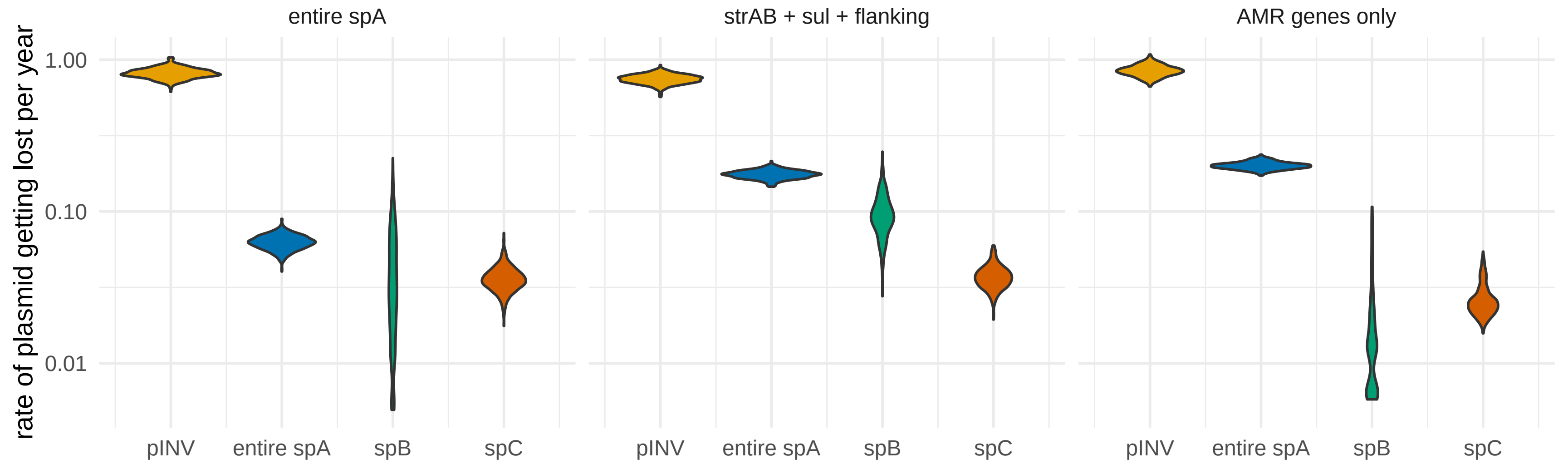

entire spA
strAB + sul + flanking
AMR genes only
rate of plasmid getting lost per year
1.00
0.10
0.01
pINV
entire spA
spB
spC
pINV
entire spA
spB
spC
pINV
entire spA
spB
spC
