# Supplementary material for: Quantifying plasmid movement in drug-resistant Shigella species using phylodynamic inference
Source: PLoS Pathog. 2025 Dec 1;21(12):e1013621. doi: 10.1371/journal.ppat.1013621 (PMC12677775; doi:10.1371/journal.ppat.1013621)
Supplement: S14 Fig — Here, we compare the posterior distribution of the number of plasmid transfer events when using different parts of the spA plasmid for inference. Each violin plot is created from a different analysis using either the entire spA plasmid, the combination of four AMR genes from sul2 to tetA, the three AMR genes sul2, strA,strB, and the flanking region of ∼100 bases. (PDF) [file ppat.1013621.s016.pdf]

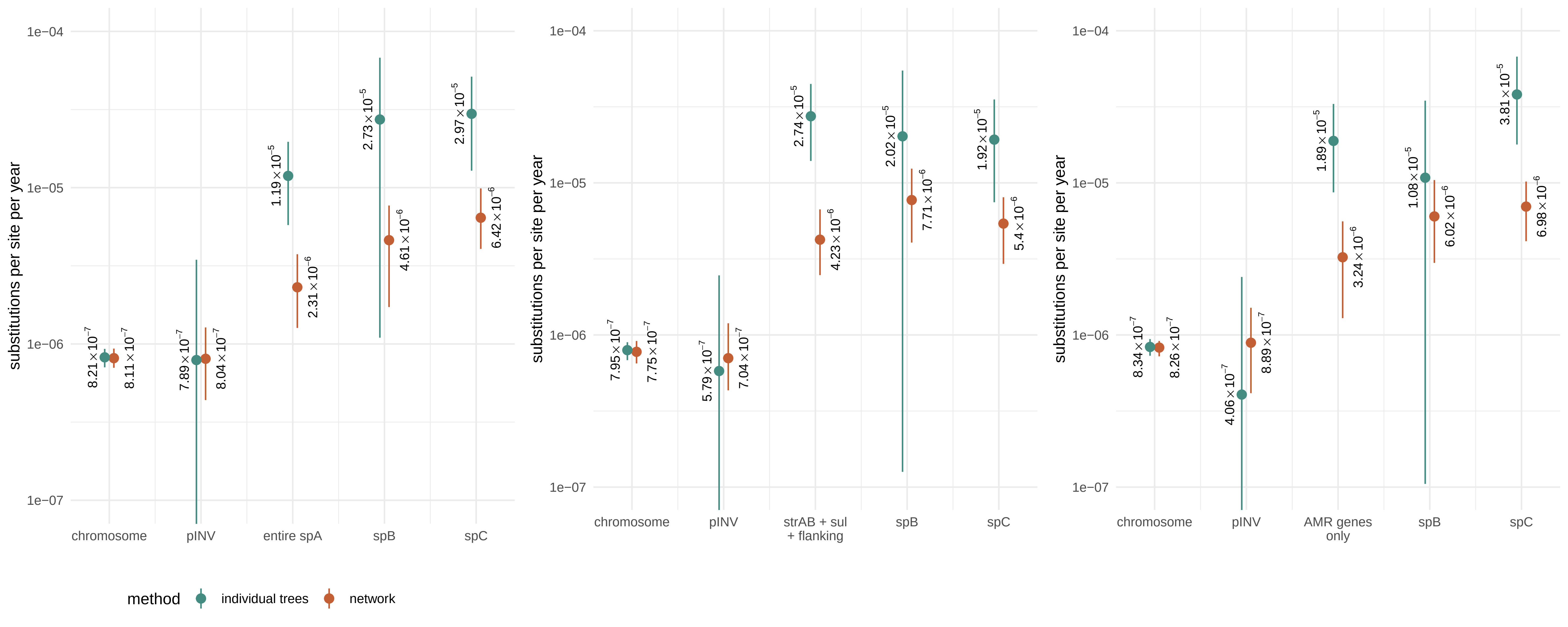
substitutions per site per year
1e−04
1e−05
1e−06
1e−07
chromosome
pINV
entire spA
spB
spC
8.21 × 10^−7
8.11 × 10^−7
7.89 × 10^−7
8.04 × 10^−7
1.19 × 10^−5
2.31 × 10^−6
2.73 × 10^−5
4.61 × 10^−6
2.97 × 10^−5
6.42 × 10^−6
strAB + sul + flanking
7.95 × 10^−7
7.75 × 10^−7
5.79 × 10^−7
7.04 × 10^−7
2.74 × 10^−5
4.23 × 10^−6
2.02 × 10^−5
7.71 × 10^−6
1.92 × 10^−5
5.4 × 10^−6
AMR genes only
8.34 × 10^−7
8.26 × 10^−7
4.06 × 10^−7
8.89 × 10^−7
1.89 × 10^−5
3.24 × 10^−6
1.08 × 10^−5
6.02 × 10^−6
3.81 × 10^−5
6.98 × 10^−6
method
individual trees
network
