# Supplementary material for: Quantifying plasmid movement in drug-resistant Shigella species using phylodynamic inference
Source: PLoS Pathog. 2025 Dec 1;21(12):e1013621. doi: 10.1371/journal.ppat.1013621 (PMC12677775; doi:10.1371/journal.ppat.1013621)
Supplement: Attachment — Submitted filename: PPATHOGENS-D-25-00988_reviewerReport.pdf [file ppat.1013621.s024.pdf]

Reviewer Report “Quantifying plasmid movement in drug-resistant Shigella species using phylodynamic inference”

The manuscript “Quantifying plasmid movement in drug-resistant Shigella species using phylodynamic inference” does a very intersesting job in trying to answer one of the most pressing questions in plasmid genomic epidemiology, namely understanding how plasmid spread across bacterial hosts. They develop a novel Bayesian approach that integrates coalescent and plasmid transfer. The apply the method to a Shigella dataset to quantify the movement of the different plasmids present.

While the methods is really interesting and well conceived, and the analysis sound, the presentation of the manuscript is below the standards expected for pubblication and would need some work.

My most important concern is on the co-divergence between plasmids and chromosome. It is a very interesting point and one of the main innovations of this work, but I think needs more detailed explanation on how it is derived and how it works (there is a long introduction on why it is needed in results, but I find it a little confusing and I think it needs tidying up). The co-divergence is not explicitly mentioned in methods, and it should be. Also a little of discussion on how it takes into account/ is affected by the huge uncertainties on how long plasmids have really spent in the host would be interesting.

Here are some minor but very important clarifications (in the order I read the paper: methods first, results after):

- The methods section itself is missing the “Methods” header which should go in lines 315-316
- could the authors provide a more “detailed” figure than the last panel in figure 1 to show how the model works (lines 328 on). Maybe with two plasmids or a slighltly complex tree.
- in line 346 the authors define μ as the parameters of the subsitution model. Can the authors add “molecular” for clarity?
- in line 374 there is a typo in the formula $t_i - 1$ should be t_{i-1} and a wrong parenthesis (square bracket is closed by a round bracket).
- in the denominator in formula (7), are the authors writing the most general expression, but it could be simplified if (as they seem to be assuming) $N_e(t)$ is constant? Maybe they should clarify this afterwards for the busy reader.
- formula 8 is not adequately explained, $\mathcal{L}_i$ is not defined.
- Maybe a supplementary picture illustrating the operators in lines 398 and following could be useful. Or, at any rate, a more detailed definition in the text: can the authors add a definition of tree length (line 405)? It is kind of intuitive, but does not hhurt to define it. same for “height of the nodes” (line 407)
- in lines 435 and following, the three plasmids spA, spB and SpC are introduced without saying they are plasmids. Again, it might be intuitive.
- the way all SNPs allignment of the chromosome has been done is really good and careful and I would like to praise the authors for putting so much care in obtaining a meaningful allignment.
- in line 474 there is the github link for the Core-SNP-filter, but a quetion mark in the citation. Either the authors fix the citation or get rid of the question mark.
- the paragraphs starting in lines 489 and 497 describe the different allignments used for the plasmids. But then in the results (lines 90, 95 and 96) the same allignments are called with different names. Can

the authors fix this?

- in my opinion supplementary figures S3 S4 S5 and S6 are relevant for the understanding of the work and should be in the main text (if the journal format allows, of course)
- the two sentences in lines 119-122 should have an equivalent in methods but I did not find it.
- the sentence in line 143 should be moved to discussion and expanded
- the paragraph starting in line 146 has again no equivalent in the methods sections, but most importantly, does not give the result. It explains how the plasmid loss rate is computed (which should probably be in methods) but does not give a value for what the plasmid loss rate thus computed is. Either fix it or remove it (removing it would be a pity because it is a novel and very interesting analysis)
- In line 166 the authore refer to Supplementary figure 3 and SF10. They have referred to Supplementary Figure through the text so SF should be changed.
- In line 191 it seems that the inferred evolutionary rates have been obtained using both the chromosome and plasmid allignments, but I am not clear how. I think it is described in methods in the section starting at line 466 and part of it is the paragraph starting in line 532. If it is, the word "co-divergence" is never used in either and it is absolutely not clear that chromosome and plasmid alignment have been used. Either way a more clear section on the "co-divergence" analysis needs to be present in methods and the sentence in line 183 needs further clarification.
- same problem for the results section starting at line 206. It needs to refer to a clearer methods section (where all the details such as the substitution model used) need to be recorded.
