# Supplementary material for: Quantifying plasmid movement in drug-resistant Shigella species using phylodynamic inference
Source: PLoS Pathog. 2025 Dec 1;21(12):e1013621. doi: 10.1371/journal.ppat.1013621 (PMC12677775; doi:10.1371/journal.ppat.1013621)
Supplement: Attachment — Submitted filename: Shigella plasmid reviews - Google Docs.pdf [file ppat.1013621.s025.pdf]

We would like to thank the reviewers for their time and thoughtful reviews that helped to improve the manuscript. We provide a detailed response to each comment below.

Reviewers' Comments:

Reviewer's Responses to Questions
Part I - Summary
Please use this section to discuss strengths/weaknesses of study, novelty/significance, general execution and scholarship.

Reviewer #1: The authors present a new Bayesian method for better understanding plasmid dynamics, which is of general interest as antimicrobial resistance (AMR) poses a major global health threat, largely because AMR genes can spread between bacteria via mobile elements like plasmids. Tracking how plasmids move and spread resistance has been difficult with current computational tools. In response, this study introduces a new Bayesian phylogenetic network method that models the co-evolution of chromosomal and plasmid DNA, enabling researchers to reconstruct and quantify plasmid movement between bacterial lineages over time while accounting for uncertainty.

Applying this approach to a five-year dataset of Shigella, the authors analyzed the transfer rates of plasmids with different resistance and virulence profiles. This method offers valuable insights into how AMR-carrying plasmids spread and persist in bacterial populations, potentially improving strategies to combat antimicrobial resistance. I have a few minor comments that should be addressed.

Reviewer #2: Muller and co-authors presented here a new phylodynamic model named CoalPT, to investigate the evolutionary dynamics of AMR-carrying plasmids in Shigella sonnei and flexneri, also starting from short-read data. This model is based on coalescent process with reassortment, parametrized to quantify the transfer and evolutionary rates of plasmids of epidemiological interest, adding novel insights about plasmid movement across species, which is usually less studied due to its complexity.
The model is explained exhaustively and the workflow of the analysis is solid. I only have a few concerns that might need clarification, which I am expressing as following.

Reviewer #3: (No Response)

Part II – Major Issues: Key Experiments Required for Acceptance
Please use this section to detail the key new experiments or modifications of existing experiments that should be absolutely required to validate study conclusions.

Generally, there should be no more than 3 such required experiments or major modifications for a "Major Revision" recommendation. If more than 3 experiments are necessary to validate the

study conclusions, then you are encouraged to recommend "Reject".

Reviewer #1: (No Response)
Reviewer #2:

1. Line 217: Did the authors include a representative number of samples x year during the random sampling? This could affect the estimation of Ne and relating coalescent process e.g. coalescent rate over time

We now better explain the sampling process, in particular, we added:

"*From the overall* S. sonnei*, we subsampled 400 isolates. For the subsampling, we sampled without replacement, using the number of available plasmid sequences per isolate as the weights. We did not perform any additional subsampling to have even sample numbers over time*."

And

"Modelling changing effective population sizes over time may put a change in the relative weight on longer or shorter networks, which can then lead to slightly different plasmid transfer rate estimates. However, this bias is expected to be small for cases with well-informed rates of evolution, such as provided by the chromosomal sequences here, and the rates across the different plasmids will be affected to a similar degree."

2. Line 139-145 The authors observed that the number of events and transfer rates was lower when using only single AMR genes compared to the AMR genes + the flanking regions or the whole plasmid. It would be expected that if genes are moving together e.g. transposons are prone to high transfer rates, being more plastic in terms of HGT compared to the whole plasmid transfer. How was the convergence of the model when using only AMR rather than the other two alignments? It would be helpful to confirm the authors's hypothesis about the absence of phylogenetic signal using dedicated tools e.g. Tempest

We now explicitly test whether the difference in results between the spA is driven by the different parts of the spA alignment coding for different histories or by more or less phylogenetic signal. To do so, we added a supplementary figure where we have run an additional analysis using the coalescent with reassortment using three 'segments': the entire spA, *str sul* genes, and the flanking region, and the 4 AMR genes only. We then estimated the number of reassortment events, which can be interpreted as there being a signal for the different alignments to code for a different evolutionary history. We did not find evidence for the different alignments to code for different evolutionary histories. As such, we attribute the results to a difference in the 'available' plasmids and the amount of genomic sequences rather than a difference in the amount of signal that originates from using different parts of the plasmid. We added a supplementary figure and now write that

We next tested whether there is evidence for the different parts of spA to code for different evolutionary histories, or whether they mostly differ by their amount of genomic information. To do so, we analysed isolates that reached the coverage thresholds for all three parts of the spA plasmid as described above. We then treated them each as a separate segment in a phylogenetic network analysis and reconstructed the joint reticulation network describing the shared evolutionary history of the three parts of spA, not finding evidence for the different parts of spA to code for different evolutionary histories (see S5 Fig)

Tempest looks at whether there is enough time signal to estimate the evolution over time from the sequences, in this case, a lot of the time information comes from the co-divergence of the plasmids with the chromosome, which cannot be captured by e.g. Tempest.

3. Line 227 The authors parametrize the model to account for different plasmid rates, and they found evidence of rapid expansion of a clade in some cases. Why did the authors not consider using alternative models other than strict e.g. fixed local when calibrating molecular clock, which allows lineages to evolve under different evolutionary rates?

The reviewer raises an interesting point. The strict clock assumption could, in theory, bias plasmid transfer inference. The assumption could falsely put weight on shorter or longer branches that could be interpreted by the model as a signal for plasmid movement. The plasmid sequence alignments themselves do not have a lot of genomic information, and hence won't have any information about relaxed clocks. I.e., most branches will have zero mutations. Further, the models are currently not computationally efficient enough to deal with relaxed clock models. However, the alignments of the chromosome may have enough genomic information. To test this we did add an analysis using chromosomal isolates to see if there is strong evidence for a relaxed clock (see supplemental figure).

Lastly, we tested whether the strict clock assumption we used for the chromosomal data was valid. We find some evidence for a higher coefficient of variation describing rate heterogeneity across lineages in the true data compared to simulated data under a strict clock, but the confidence intervals overlap (S15 Fig). This indicates that there may be slight variation in evolutionary rates among lineages that are not fully accounted for using the strict clock model.

Reviewer #3: (No Response)

Part III – Minor Issues: Editorial and Data Presentation Modifications
Please use this section for editorial suggestions as well as relatively minor modifications of

existing data that would enhance clarity.

Reviewer #1:

1. "Methods" section title missing

This has been added.

2. line: 333/4 : "In fact, it is the backward-in-time equivalent of one plasmid being transferred between bacterial lineages at a time." I don't understand what this equivalence refers to, please clarify.

We now rephrased to: *"In other words, at each plasmid transfer event, we say one plasmid has originated from a different parental lineage than the chromosome. The backwards-in-time rate of a plasmid having originated from a different parental lineage models the forward-in-time process where exactly one plasmid moves from one bacterium to another."*

3. Please clearly state and discuss the independence assumption on events (plasmid transfer, coalescent, sampling events)

We have now added to the model description as follows:

"As is in coalescent approaches on trees, we condition on the sampling event, meaning that we assume the sampling events occur at predefined times. As such, the assumptions of the coalescent process apply, such as that a very small fraction of the overall population is sampled. The assumption applies on the isolate level. On the level of plasmids and chromosomes, we assume that for each type of plasmid, we have one sample per isolate. For isolates for which we do not have a specific version of a plasmid, we treat that plasmid as missing information, rather than explicitly modelling its absence. The coalescent events are assumed to happen independently of plasmid transfer events. In particular, this assumes there are no couple of coalescent/plasmid transfer events, which become more likely for very small population sizes [46]. "

4. Please discuss what assumptions are made for the sampling process - very low sampling proportion for both chromosome and plasmids? How realistic are these here and how much may that affect results? This could be addressed via simulations, but a thorough discussion would also suffice.

The reviewer makes an important point. The assumptions about sampling process are the same as those of the standard coalescent. We explained this point in the Methods section:

Under a coalescent process sampling intensity is generally assumed to be low, relative to the effective population size (a function of θ here) [46]. This assumption applies to the sampling at the level of the isolates in our model. Each isolate is assumed to have at least the chromosomal sequence. In cases where isolates do not have the full set of plasmids, the model currently

treats the plasmids as missing data rather than explicitly modelling the loss of plasmids. However, an important distinction is that even when the plasmid transfer and coalescent parameters are correctly estimated, the number of plasmid transfer events inferred refers to those that explain the samples collected, and not necessarily those of the whole population, a situation that is analogous to discrete phylogeographic models [47].

5. Fig 2 "Proportion of lineages carrying a plasmid between 2000 and 2020." How much does this result depend on sampling scheme? And how does it compare to occurrence data? Could the method also include occurrence data =?

We now clarify in the caption of Figure 2 that the dashed lines correspond to the proportion of all isolates in the complete dataset with a plasmid, which is the closest to occurrence data we have available.

6. Fig 2 "The text denotes the posterior probability of plasmid transfer events for events with a posterior support of over 0.5. " What text does this refer to? Please fix and clarify.

The text was there in error, and we have removed it.

7. Fig 2 "Branches are colored to denote separate clades originating from individual jumps of plasmids into a bacterial clade. " Which colours does this refer to? Please fix and clarify.

The text was still there in error from a previous version of the figure, and we have removed it.

8. p.8 Please explain the choice of thresholds for the input alignments (80% plasmid coverage, 95% core conservation, 250 subsamples)

We have provided details in the methods subsection '*Generation of plasmid alignments for* S. sonnei *and* S. flexneri'. We have amended the text in the results section to provide clearer directions to the reader for details. The revised text is below.

All S. sonnei and S. flexneri isolates that had ≥80% coverage of the reference plasmid were included in the alignment and a core SNP alignment with ≥95% conservation of sites (S16 Fig)(see Methods for plasmid threshold selection for alignments). We then randomly sub-sampled 250 isolates equally from S. sonnei and S. flexneri that carried the pKSR100-like plasmids (250 isolates were selected due to computational limitations).

9. Discussion: Please state clearly if applicable to plasmid movements between completely different bacteria. Even in a structured model, this seems difficult if a joint alignment is required.

We included a sentence in the Discussion to explain this point.

“There is extensive evidence of plasmid movement between bacteria of distant lineages or species. Currently, the movement of plasmids between different species can be modelled by combining different chromosomal alignments into a single alignment, as we have done here for S. sonnei and S. flexneri to reconstruct the movement of pKSR100 between the two species. However, we had to assume a shared coalescent process. Future work should explore these processes, potentially using a hierarchical network modelling structure similar to the multispecies coalescent.”

Reviewer #2:

1. In Fig.2, how do the authors explain the scattered distribution of the plasmid on terminal tips? Would it be possible that those plasmids are prone to be lost by the strains when cultured, rather than actual loss for other reasons e.g. environmental selective pressures? The authors should discuss this potential issue

We have now added an additional analysis where we model the plasmids as present or absent using a continuous-time Markov chain. The new text now reads as:

This was expected, given the known loss in culture. To further test this point, we performed an analysis where we modeled the presence and absence of pInv, spA, spB, and spC as a continuous-time Markov chain evolving on the chromosome. We compare the rate at which plasmids are gained and lost between the true observed data and when we randomly permutate the tip to presence-absence mapping. We find that the rate of gain/loss for pInv is equal between the true and the permuted presence absence (see S11 Fig), indicating that the plasmid is randomly lost during the sampling process, while the three small plasmids show strong differences between the true presence absence and the permuted assignments.

2. Line 474 Core-SNP-filter github reference can be replaced with the publicated paper https://doi.org/10.1099/mgen.0.001346

We updated the reference.

3. Fig. S6, S7, S9, S11 It would be easy to visualize the differences if the plots were shown using only one row

We have changed these supplementary figures to have one row of plots in each.

4. Fig S3,S5 please increase label size

We have changed these supplementary figures to increase the font size.

Reviewer #3: (No Response)

Reviewer 3

The manuscript “Quantifying plasmid movement in drug-resistant Shigella species using phylodynamic inference” does a very intersesting job in trying to answer one of the most pressing questions in plasmid genomic epidemiology, namely understanding how plasmid spread across bacterial hosts. They develop a novel Bayesian approach that integrates coalescent and plasmid transfer. The apply the method to a Shigella dataset to quantify the movement of the different plasmids present.

While the methods is really interesting and well conceived, and the analysis sound, the presentation of the manuscript is below the standards expected for pubblication and would need some work.

1. My most important concern is on the co-divergence between plasmids and chromosome. It is a very interesting point and one of the main innovations of this work, but I think needs more detailed explanation on how it is derived and how it works (there is a long introduction on why it is needed in results, but I find it a little confusing and I think it needs tidying up).

We thank the reviewer for the positive comments on the interest and innovation of this work, and for raising that we needed to expand upon the co-divergence between different plasmids and the chromosome in this point, and in point 2 and point 20. We have added new text to address the reviewers concerns to more fully explain the approach in the methods. We have provided details in points 2 and more fully in point 20 below. Further, we reviewed the text in the results that describes CoalPT and the resulting output of timed phylogenetic networks, and decided to it was important to keep at the start of the results in addition to the new text that has been added to the methods.

2. The co-divergence is not explicitly mentioned in methods, and it should be.

We added several sentences about co-divergence in the Methods, specifically in *Accounting for the co-divergence of chromosomes and plasmids is essential to estimating rates of evolution in plasmids* subsection. We have focused in particular on the estimation of evolutionary rates of plasmids where coalescent events where chromosomes and plasmids co-diverged act essentially as calibration points in time. This is fully detailed below on co-divergence for point 20 raised by the reviewer.

3. Also a little of discussion on how it takes into account/ is affected by the huge uncertainties on how long plasmids have really spent in the host would be interesting.

The reviewer raises an interesting point. We have added some text in the Discussion to highlight that estimating the length of time plasmids have spent co-evolving with their hosts is an area that this work could be expanded upon in the future. The new text now reads:

Finally, there are uncertainties around estimating the length of time some plasmids may have spent with their hosts. However, this approach provides the basis that these methods may be further refined to better capture the impacts of different plasmids in shaping bacterial populations.

We have also added new text and analyses in response to Review 2 point 1 (above) that relates to plasmid loss.

Here are some minor but very important clarifications (in the order I read the paper: methods first, results after):

4. The methods section itself is missing the “Methods” header which should go in lines 315-316

This has been added.

5. could the authors provide a more “detailed” figure than the last panel in figure 1 to show how the model works (lines 328 on). Maybe with two plasmids or a slighltly complex tree.

We added an additional plasmid and now show a more complex network scenario in Figure 1.

6. in line 346 the authors define μ as the parameters of the subsitution model. Can the authors add “molecular” for clarity?

This has been added.

7. in line 374 there is a typo in the formula ti−1 should be ti−1 and a wrong parenthesis (square bracket is closed by a round bracket).

We removed this part as it unnecessarily added complexity.

8. in the denominator in formula (7), are the authors writing the most general expression, but it could be simplified if (as they seem to be assuming) Ne(t) is constant? Maybe they should clarify this afterwards for the busy reader.

We switched to Ne instead of Ne(t) throughout.

9. formula 8 is not adequately explained, Li is not defined.

We have added text to explain formula 8 and to define Li. The new text now reads as:

with ni being the number of plasmids on Li and Li existing in that interval. What the above equation means is that we can observe a plasmid transfer event only on lineages that have

either the chromosome or more than one plasmid present, as we can only observe the movement of plasmids relative to other plasmids or the chromosome.

10. Maybe a supplementary picture illustrating the operators in lines 398 and following could be useful. Or, at any rate, a more detailed definition in the text: can the authors add a definition of tree length (line 405)? It is kind of intuitive, but does not hurt to define it. same for "height of the nodes" (line 407)

We added a figure to better explain the add and remove operator, the main operator, as well as explanations on heights.

11. in lines 435 and following, the three plasmids spA, spB and SpC are introduced without saying they are plasmids. Again, it might be intuitive.

We have added text in the *Reference genomes* subsection of the Methods to make clear that spA, spB and spC are plasmids.

12. the way all SNPs allignment of the chromosome has been done is really good and careful and I would like to praise the authors for putting so much care in obtaining a meaningful allignment.

We thank the reviewer for the positive comments.

13. in line 474 there is the github link for the Core-SNP-filter, but a quetion mark in the citation. Either the authors fix the citation or get rid of the question mark.

This has been edited to fix the citation.

14. the paragraphs starting in lines 489 and 497 describe the different allignments used for the plasmids. But then in the results (lines 90, 95 and 96) the same allignments are called with different names. Can the authors fix this?

In the methods section we have described how each of the alignments were generated. We have included the name of the alignments reported in the methods in brackets so it is clear which method relates to which alignment.

15. in my opinion supplementary figures S3 S4 S5 and S6 are relevant for the understanding of the work and should be in the main text (if the journal format allows, of course)

We have chosen to leave these four supplementary figures in the supplementary information given we already have five main figures in the manuscript and would need to replace one of the figures.

16. the two sentences in lines 119-122 should have an equivalent in methods but I did not find it.

We have added text to the Methods in the *Coalescent with plasmid transfer* that includes the clock and substitution models.

17. the sentence in line 143 should be moved to discussion and expanded

We have moved and edited this sentence. It now sits in the Discussion and reads as:

We demonstrated the utility of this novel method for the reconstruction of the movement of plasmids but also showed the limitation of this approach, particularly for small AMR mobile elements, where there may be a lack of phylogenetic signal. As such, it is not suited for tracking the movement of specific AMR genes within populations in the absence of any other phylogenetic context.

18. the paragraph starting in line 146 has again no equivalent in the methods sections, but most importantly, does not give the result. It explains how the plasmid loss rate is computed (which should probably be in methods) but does not give a value for what the plasmid loss rate thus computed is. Either fix it or remove it (removing it would be a pity because it is a novel and very interesting analysis)

We have added text in the Methods that clarifies how the rate of plasmid loss was determined in *S. sonnei.* This new text now reads as:

We next computed the rate at which plasmids are being lost in S. sonnei. We first calculated the number of times a plasmid has been lost as the number of child edges (i.e., branches) in a network for which the parent branch carries a plasmid while the child branch itself does not. We then divide this number by the total length of the plasmid tree to get an estimate of the rate at which the plasmid is lost in units of plasmid loss events per unit time. We restrict this analysis to events in the last 10 years since sample collection. We calculate this heuristic for the posterior distribution of networks to estimate the uncertainty of the heuristic. Importantly, the rate at which plasmids are lost is a heuristic based on the reconstruction networks and not directly a model parameter. In the future, this rate could be explicitly incorporated as a model parameter into CoalPT.

Further, we have undertaken additional discrete trait analyses of the presence/ absence of the plasmid profiles, writing that

"To further test this point, we performed an analysis where we modeled the presence and absence of pInv, spA, spB, and spC as a continuous-time Markov chain evolving on the chromosome. We compare the rate at which plasmids are gained and lost between the true, observed data, and when we randomly permutate the tip to presence-absence mapping. We find that the rate of gain/loss for pInv is equal between the true and the permuted presence

absence (see S11 Fig), indicating that the plasmid is randomly lost during the sampling process, while the three small plasmids show strong differences between the true presence absence and the permuted assignments. We also estimated the individual rates of gain and loss using this approach (see S12 Fig). While this doesn't fully model the ancestral history of each plasmid, we find that the estimates are broadly consistent with both our estimates using the phylogenetic network inference."

19. In line 166 the authore refer to Supplementary figure 3 and SF10. They have referred to Supplementary Figure through the text so SF should be changed.

We have edited the text to refer to the supplementary figures consistently throughout, and using the PLoS style e.g. S1 Fig.

20. In line 191 it seems that the inferred evolutionary rates have been obtained using both the chromosome and plasmid allignments, but I am not clear how. I think it is described in methods in the section starting at line 466 and part of it is the paragraph starting in line 532. If it is, the word "co-divergence"is never used in either and it is absolutely not clear that chromosome and plasmid alignment have been used. Either way a more clear section on the "co-divergence" analysis needs to be present in methods and the sentence in line 183 needs further clarification.

We added the terms co-divergence and calibration points to the description and provided more details on how the analyses were performed

That is the measurable accumulation of genomic change over the time window of sampling. However, modelling the joint evolution of chromosomes, which are measurably evolving, and plasmids changes this. The timing of past coalescent events where chromosomal and plasmid co-diverged is potentially well informed by the chromosomal DNA, which is measurably evolving. These 'co-divergence' events of chromosomes and plasmids can then act as calibration points to infer the rates of evolution of plasmids.

And

We used the same priors and evolutionary models (strict clock model and HKY+Γ_4) as for the network inference described above and a constant coalescent population model. We then inferred the phylogenetic trees, evolutionary rates, and other parameters. For the network inference, we inferred a separate evolutionary rate for the chromosome and each plasmid. To calculate from the SNP alignment to the whole genome rate of evolution of the chromosome and the virulence plasmid, we multiply the rate estimate based on the SNP alignment by the ratio of SNP sites (11kb) over the whole genome length (4.8Mb).

21. same problem for the results section starting at line 206. It needs to refer to a clearer methods section (where all the details such as the substitution model used) need to be recorded.

We have added text to the Methods in the *Coalescent with plasmid transfer* that includes the clock and substitution models.

Reviewer # 4

The authors introduce a coalescent model that includes an additional plasmid conjugation process. The model is used to analyze the plasmid transfer rates in Shigella and could help to understand the spread of plasmids with AMR conferring genes.

Overall I enjoyed reading this manuscript and analyzing plasmid transfer in larger datasets based on solid models is a highly relevant topic of interest. The Bayesian framework presented here is an important step in that direction.

However, I was missing a precise and more detailed description of the model used in this work and how it was exactly applied to the datasets. For example, for equation 8 it would be essential to have a supplemental file that explains the calculations in more detail and illustrates the process.

We have now expanded on the description of when we can observe the movement of plasmids in the main text by writing. We also clarified equation 9 itself:

What the above equation means is that we can observe a plasmid transfer event only on lineages that have either the chromosome or more than one plasmid present, as we can only observe the movement of plasmids relative to other plasmids or the chromosome.

In the current manuscript it does for example not get clear, whether two lineages with only the plasmid can coalesce with each other or not and whether lineages with multiple different plasmids can split again via plasmid transfer. The current notation would allow both to be true and Figure 1 does also not include this scenario (which is possibly fine to keep the concept figure simple, but it should be clarified what the actual process does somewhere.).

We changed Figure 1 to show a more complex scenario that includes the case where two lineages that only carry the plasmid can coalesce. The method allows for any network topology that can be simulated using the coalescent with plasmid transfer.

Line 363: It is not clear to me whether the authors used BEAST2 as a tool or re-implemented the same algorithms for their own analysis.

We now state in the intro more clearly that:

Our method is implemented as a package for the open-source software BEAST2 to facilitate its adoption. As such, one can use the evolutionary models already implemented in BEAST2.

And in the operator description:

For parameter inference, such as the inference of the N_e and rho or the evolutionary parameters, we use the operators already implemented in BEAST2.

Minor comments:

It might be worth explaining somewhere what a maximum clade credibility network is.

We added to the caption in Figure 1 that:

The MCC network is the network in the posterior distribution that maximizes the sum of clade credibility of the chromosome and plasmid trees.

The heading for the Materials and Methods section is missing.

We added the header to the updated version.

There seems to be some confusion around the parameter k in the first section of Materials and Methods (lines 316 – 330). At one point k seems to be the number of all lineages at a later point k is the number of lineages that have both the chromosome and the plasmid. Certainly both can not be true at the same time and lineages with only plasmids still need to coalesce with at least some other lineages.

We cleaned up that part of the methods to be consistent.

Line 363: allow us to (-missing word?-) all the default …

Thank you for noticing. We fixed the error.

Figure 4: The caption claims that the figure shows the posterior estimate of the plasmid transfer rate, but no rate is shown. Also if it would be the distribution of the number of events along a certain time window, for a poisson process a bimodal distribution would not occur. Maybe this is for a given specific tree? Can you explaNe(t_i) in what exactly the figure is showing in the manuscript?

We completely changed the figure description of Figure 4. The revised text now reads as:

Here, we show the posterior estimates for the number of times where a plasmid moved between S. sonnei and S. flexneri. The assignment of directionality is based on the chromosome. For

example, if the parental node at a plasmid transfer event has a chromosome belonging to S. sonnei, the origin is considered S. sonnei.

Equation 4: Ne(t_i) would be less confusing using N_e (t_i) instead ?

We changed to N_e throughout and also moved to denote the Network as 'mathcal' N instead of N to more clearly distinguish between the two.

Line 146 – 152: Is the rate at which plasmids are lost based on one specific estimated tree or considered in a Bayesian framework?

We now clarify the section by expanding the text as follows:

We next computed the rate at which plasmids are being lost in S. sonnei. We first calculated the number of times a plasmid has been lost as the number of child edges (i.e., branches) in a network for which the parent branch carries a plasmid while the child branch itself does not. We then divide this number by the total length of the plasmid tree to get an estimate of the rate at which the plasmid is lost in units of plasmid loss events per unit time. We restrict this analysis to events in the last 10 years since sample collection. We calculate this heuristic for the posterior distribution of networks to estimate the uncertainty of the heuristic. Importantly, the rate at which plasmids are lost is a heuristic based on the reconstruction networks and not directly a model parameter. In the future, this rate could be explicitly incorporated as a model parameter into CoalPT.

Figure 2:
I do not understand this part of the caption:
The dashed lines correspond to branches from which plasmids branch off. The text denotes the posterior probability of plasmid transfer events for events with a posterior support of over 0.5. Branches are colored to denote separate clades originating from individual jumps of plasmids into a bacterial clade.I can not see dashed lines and do not know which text you are referring to. Also the color of the plasmid transfer branches seems to be just the color corresponding to the plasmid.

That part was there by accident, and we removed it.

Figure 2: panels B and C are actually one panel and not two separate panels.

We removed panel C from Figure 2 and reduced the size of the labels.

Figure 5: The legend in part A (<0, 1, 2) does not make sense to me. How can the number be negative, and what is the difference between the lines?

We fixed the legend in part A and added the A label to it. The possibility for negative values is due to the values denoting log standardized values.

Figure resubmission:

While revising your submission, please upload your figure files to the Preflight Analysis and Conversion Engine (PACE) digital diagnostic tool, https://pacev2.apexcovantage.com/. PACE helps ensure that figures meet PLOS requirements. To use PACE, you must first register as a user. Registration is free. Then, login and navigate to the UPLOAD tab, where you will find detailed instructions on how to use the tool. If you encounter any issues or have any questions when using PACE, please email PLOS at figures@plos.org. Please note that Supporting Information files do not need this step. If there are other versions of figure files still present in your submission file inventory at resubmission, please replace them with the PACE-processed versions.

Reproducibility:

To enhance the reproducibility of your results, we recommend that authors of applicable studies deposit laboratory protocols in protocols.io, where a protocol can be assigned its own identifier (DOI) such that it can be cited independently in the future. Additionally, PLOS ONE offers an option to publish peer-reviewed clinical study protocols. Read more information on sharing protocols at https://plos.org/protocols?utm_medium=editorial-email&utm_source=authorletters&utm_campaign=protocols
